# Extracellular matrix stiffness reduces DNA 6 ma level to facilitate colorectal cancer progression via disrupting P53 binding to CDKN1A promoter

**DOI:** 10.1186/s40164-025-00704-w

**Published:** 2025-08-27

**Authors:** Si-An Xie, Xue Li, Min-Yue Yin, Feng Du, Shu-Tian Zhang, Sheng-Tao Zhu

**Affiliations:** 1 Department of Gastroenterology, Beijing Friendship Hospital, Capital Medical University, Beijing, China; 2 National Clinical Research Center for Digestive Disease, Beijing Digestive Disease Center, State Key Laboratory of Digestive Health, Beijing, 100050 China

**Keywords:** Matrix stiffness, DNA 6 ma modification, ALKBH1, Colorectal cancer, p53, CDKN1A

## Abstract

The extracellular matrix (ECM) forms the primary scaffold of the tumor microenvironment, with matrix stiffness serving as a critical physical cue that modulates cancer progression. However, the impact of matrix stiffness on colorectal cancer (CRC) progression remains elusive. This study aimed to elucidate the role of substrate stiffness in regulating DNA N6-methyladenine (6 mA) modifications and their association with CRC progression. We observed significantly reduced DNA 6 mA levels in CRC cells and tissues compared to normal controls, which progressively declined with advancing CRC stages. A negative correlation was identified between CRC tissue stiffness and DNA 6 mA levels. The 6 mA demethylase ALKBH1 was identified as a poor prognostic indicator in CRC and responded to increased substrate stiffness, correlating with enhanced CRC proliferation. Mechanistically, ALKBH1 mediated DNA 6 mA demethylation in response to substrate stiffening, thereby modulating gene transcription and promoting CRC tumorigenesis. Notably, ALKBH1 lost its proliferative effect in P53-knockout CRC cells, while a catalytically inactive ALKBH1 mutant suppressed oncogenesis. Furthermore, ALKBH1 diminished CDKN1A expression by impairing P53 binding to the CDKN1A promoter region. Collectively, our findings demonstrate that ALKBH1 acts as a pivotal mediator linking matrix stiffness to DNA 6 mA demethylation, critically driving CRC progression and highlighting its therapeutic potential. These results underscore the importance of DNA 6 mA modifications in CRC development and tumor response to microenvironmental cues.

**Supplementary Information:**

The online version contains supplementary material available at 10.1186/s40164-025-00704-w.

## Introduction

Colorectal cancer (CRC) stands out as one of the most prevalent malignant tumors and a leading cause of cancer-related mortality on a global scale [1, 2]. Gaining a comprehensive understanding of the mechanisms underpinning the occurrence and development of CRC is crucial for enhancing diagnostic sensitivity and improving the survival rate of CRC patients. The extracellular matrix (ECM), a key non-cellular component of the tumor microenvironment, furnishes biophysical and biochemical support. It plays a pivotal role on tumor growth, angiogenesis, metastasis, immune evasion, and drug resistance, thus playing a pivotal role in tumor biology [3–5]. Notably, CRC tumor stiffness is markedly elevated compared to normal tissues and exhibits a positive correlation with tumor progression [6]. Mechanical signals stemming from matrix stiffness can be translated into biological signals via mechanical sensors or transducers to regulate tumor-associated gene expression [7–10]. For instance, targeted reversal of matrix stiffness in pancreatic ductal adenocarcinoma has been shown to effectively enhance the sensitivity of chemotherapy-resistant tumors [11]. Unraveling the molecular mechanisms of matrix stiffness holds great significance for prognostic assessment and therapeutic intervention in CRC. Nevertheless, the precise means by which matrix hardness influences CRC-related gene expression through epigenetic modifications remains a vast and underexplored research frontier.

DNA methylation modifications, encompassing 5-methylcytosine (5mC) and its oxidized derivatives as well as N6-methyladenine (6 mA), are well-recognized epigenetic modifications that modulate gene expression [12, 13]. 5mC modification involves methylation or demethylation at the 5-position of cytosine and is predominantly found in palindromic CpG dinucleotides [14]. While 5mC is a well-established epigenetic mark critical for transcriptional silencing, genomic stability, and development in mammals, the functional significance and regulatory mechanisms of 6 mA in mammalian transcription are still not fully understood and remain a topic of intense debate [14]. Recent technological advancements in 6 mA detection and sequencing have ushered in new insights into 6 mA biological research in mammals [15]. DNA 6 mA modification is primarily associated with the [G/C]AGG[C/T] motif and is significantly enriched in the promoter and exon region, where it activates gene expression [16]. Generally, DNA 6 mA levels are lower in tumor tissues than in normal tissues, as observed in liver cancer, lung cancer, and hepatocellular carcinoma [17, 18]. Research on DNA 6 mA in eukaryotic organisms is still in its infancy, with current research efforts primarily focusing on 6 mA-related methylases and demethylases [19]. AlkB homolog 1, histone H2A dioxygenase (ALKBH1), has emerged as a critical DNA 6 mA demethylase. It contains a conserved Fe^2+^ and 2-oxoglutarate-dependent dioxygenase domain, which serves as the central active region [18]. Elevated expression of ALKBH1 has been implicated in promoting several types of tumors, including gastric cancer, lung cancer and head and neck squamous cell carcinoma [20–23]. Despite these findings, whether the demethylase ALKBH1-mediated DNA 6 mA modification regulates CRC progression remains an unexplored area.

In this study, we investigated the phenomenon that DNA 6 mA levels progressively decrease as CRC develops. We found that increased expression of the demethylase ALKBH1 in response to stiffness stimulation leads to reduced DNA 6 mA modification. Mechanistic exploration revealed that high ALKBH1 levels inhibit the expression of the tumor suppressor gene CDKN1A by blocking P53 binding to the CDKN1A promoter and suppressing CDKN1A transcriptional activity. Thus, ALKBH1-driven DNA 6 mA demethylation is upregulated under ECM stiffness stimulation, representing a novel epigenetic modification in response to the tumor microenvironment that exacerbates CRC.

## Materials and methods

### Cell culture

Human CRC cell lines (HCT116, HCT15, HT29, RKO, and SW480) and human colorectal normal epithelial cells (NCM460) were purchased from the Cell Bank of Type Culture Collection of Chinese Academy of Sciences, Shanghai Institute of Biochemistry and Cell Biology. HCT116 was cultured in McCoy’s 5a medium containing 10% fetal bovine serum and penicillin (100 U/ml)/streptomycin (0.1 mg/ml). HCT15, HT29, RKO, SW480, and NCM460 cell lines were cultured in Dulbecco’s modified Eagle’s medium containing 10% fetal bovine serum and penicillin (100 U/ml)/streptomycin (0.1 mg/ml). All cell lines applied in the experiments were performed less than three passages after cell thawing.

### Lentivirus preparation, plasmids and cell transfection

The shALKBH1 was constructed into pLKO.1 lentiviral vector. These plasmids were co-transfected into the 293T cells with the packaging by the standard calcium chloride transfection (lentivirus: packaging plasmid: envelope plasmid = 2:1:1). After 48 h, lentiviral supernatants were collected and concentrated by ultracentrifugation or PEG4000 + centrifugation and stored at − 80°C. The target sequence of short hairpin RNAs was as follows: shALKBH1-1, 5’-GUGAUCAAAUCUCAGCUAA-3’, shALKBH1-2, 5’-GGACA GATCTGAGCTAGAT-3’, and shNC, 5’-TTCTCCGAACGTGTCACGT-3’. Cells (HCT116 and RKO) were infected and selected with 2 mg/ml puromycin for 2 days. After selection, cells were used in the following experiments. To overexpress ALKBH1 in CRC cells, the plasmids expressing a C-terminal flag tag on the protein of ALKBH1 (ALKBH1-flag) or plasmid vectors (PV) were generated from GENEWIZ Technology. The production process of ALKBH1 enzyme activity mutant plasmids is as described in the previous article [20, 24]. In brief, a significant structural feature of hALKBH1 at the N-terminus is the Flip0 (residues 19–32). R24A/K25A/R28A/R31A in Flip0 domain completely lost demethylation activity toward 6 mA ssDNA. The arginine or lysine at positions 24, 25, 28 and 31 was mutant to alanine. Based on the overexpression plasmid of ALKBH1, the ALKBH1 mutant plasmid was constructed using the double-point mutation kit from NOVAZEN. The transfection of plasmid was performed using X-tremeGENE HP DNA Transfection Reagent according to the manufacturer’s instructions. Following treatment was administered 2 days after the transfection. Cells then were collected for further analysis.

### Polyacrylamide (PA) gel manufacturing

PA-gels of varying stiffness were prepared as previously described [25]. 40% acrylamide and 2% bis-acrylamide stock solutions were mixed to prepare stiff or soft PA-gel solutions according to the protocol [26]. Ammonium persulfate (10%) and N, N, N’, N’-tetramethylethylenediamine (TEMED) were added to initiate polymerization. The gels were activated with sulfo-SANPAH (Pierce, 0.5 mg/ml in PBS) under UV light (264 nm) to cross-link extracellular matrix proteins onto the gel surface. The gels were then coated with collagen Type I (0.1 mg/ml, Corning) for 1 h at room temperature.

### Human tumor samples

For immunohistochemistry (IHC) staining, we utilized a human CRC tissue microarray (HColAde075Pre-01 and OD-CT-DgCol04-003) sourced from Outdo Biotech Company (Shanghai, China). Additional human CRC tumor tissues were collected from patients undergoing endoscopy at the Endoscopy Center of Beijing Friendship Hospital, with the study being approved by the Institutional Ethics Committee of Beijing Friendship Hospital (approval number 2020-P2-290-01). Clinical information is presented in Table S1.

### Animal studies

C57BL/6J-WT (aged 6–8 weeks), C57BL/6J-Apc ^Min/+^ mice (aged 6–8 weeks) and BALB/c nude mice (aged 5–6 weeks) were obtained from Beijing Vital River Laboratory Animal Technology Co., Ltd. and housed under specific-pathogen-free conditions at the Capital Medical University’s animal facility. Three CRC mice models, carcinogen azoxymethane (AOM)/ dextran sodium sulfate (DSS)-induced, transgenic Apc^Min/+^ and orthotopic xenograft model, were established in this study. (1) The AOM/DSS-induced CRC model, mice were earmarked and randomly assigned to groups by an independent individual. C57BL/6J mice of matching age were employed for the CRC model. Mice received an intraperitoneal (i.p.) injection of 7.4 mg/kg AOM. They underwent three cycles of 3% DSS for 7 days during 2, 5, and 8 weeks, and were sacrificed in week 16. (2) The transgenic Apc ^Min/+^ model, Apc^Min/+^ mice were started on a high-fat diet at the age of 10 weeks and were sacrificed and samples were collected at 26 weeks. (3) The orthotopic xenograft model, MSI-high type CRC cell line MC38 (5 × 10^5^ cells in 10 µL Matrigel per mouse) was orthotopically implanted into the rectum of male C57BL/6J-WT mice aged 6 weeks.

For BAPN treated mouse model, the DSS/AOM induced CRC mice and the orthotopic xenograft mice were randomly divided into BAPN-, and PBS-treatment groups. BAPN (50 mg/kg) was administered intraperitoneally every two days for four weeks in the BAPN group. For the xenograft model, approximately 2 × 10^^6^ HCT116 cells suspended in PBS buffer mixed with Matrigel (volume ratio 4:1) were subcutaneously injected into BALB/c nude mice. Tumor dimensions were monitored every three days, and when tumors reached a length greater than 1 cm, mice were sacrificed, and tumors were harvested for further analysis.

### Antibodies, chemicals and commercial kits

Anti-6 mA antibody (202003) was purchased from Synaptic Systems. Anti-Ki67 (ab15580) antibody was purchased from Abcam. Anti-PCNA antibody (10205-2-AP), Anti-ALKBH1 antibody (27973-1-AP), Anti-N6AMT1 antibody (16211-1-AP), and Anti-GAPDH antibody (10494-1-AP) were purchased from Proteintech. Anti-METTL4 antibody (A25600) was purchased from ABclonal. Normal rabbit IgG (#2729), Anti-CDKN1A antibody (#2947), Anti-P53 antibody (#9282), Anti-p-P53 antibody (#9284), Anti-vimentin antibody (#3932) and Anti-E-Cadherin antibody (#3195) were purchased from Cell Signaling Technology. β-Aminopropionitrile (BAPN) (HY-Y1750) was purchased from MedChemExpress.

### IHC staining assays

Tissues for IHC were fixed in 4% paraformaldehyde for 24 h, dehydrated through a series of alcohol solutions, and embedded in paraffin. Slides were incubated at 65℃ for 2 h, deparaffinized in xylene, and rehydrated in alcohol. Antigen retrieval was performed under high pressure, and endogenous peroxidase activity was quenched with 3% H_2_O_2_ for 10 min. Sections were blocked with goat serum for 1 h before overnight incubation at 4℃ with the primary antibody. After incubation with a secondary antibody for 1.5 h at room temperature, tissues were developed using a diaminobenzidine (DAB, ZLI-9019, ZSGB-BIO) kit and counterstained with hematoxylin. The IHC score was determined based on the intensity and extent of staining. Staining intensity was scored as 1 (no staining), 2 (weak), 3 (moderate), or 4 (strong). The extent of staining was scored as 1 (0%), 2 (1–25%), 3 (26–50%), 4 (51–75%), or 5 (76–100%). The final IHC score was calculated by multiplying the intensity and extent scores.

### 6 mA Dot blot and Western blot

The DNA dot blot was performed as previously reported [25]. Briefly, equal amounts of DNA were denatured with 0.4 M NaOH and 10 mM EDTA at 95℃ for 10 min, then neutralized with 2 M ammonium acetate (pH 7.0). Denatured DNA samples were spotted onto a nitrocellulose membrane and baked at 80 °C for 2 h. For western blot, equal amounts of protein were separated by 10% SDS-PAGE and transferred to polyvinylidene fluoride membranes. After blocking non-specific binding with 5% skimmed milk in TBS-T, membranes were incubated with primary antibodies diluted at 1:1000 overnight at 4℃, followed by incubation with specific secondary antibodies for 2 h at 37℃. Protein bands were visualized using Bio-Rad gel imaging system.

### 6 mA ELISA

Genomic DNA was extracted from human and murine CRC specimens, as well as cultured cell lines, using the QIAamp DNA Mini Kit from Qiagen. DNA 6 mA methylation levels were quantified using the Methyl Flash 6 mA DNA Methylation ELISA Kit (Colorimetric) following the manufacturer’s instructions. The methylated fraction of 0.1 ug DNA was recognized by a 6 mA antibody and quantified via an ELISA-like reaction. The percentage of 6 mA (6 mA%) was calculated from the optical density (OD) value using the provided formula. Methylated and unmethylated DNA served as positive and negative controls, respectively.

### RNA isolation and quantitative RT-PCR (qRT-PCR)

Total RNA was extracted using TRIZOL and reverse transcribed as recommended. qRT-PCR was performed on Q7 real-time thermal cycler with specific primers (Table S2). mRNA expression was normalized to GAPDH and relative expression was determined using the 2^−ΔΔCt^ method.

### CCK8 assays

Approximately 2000 cells were seeded into each well of a 96-well plate. At various time points post-seeding, wells were refreshed with a medium containing 10% CCK8 substrate and incubated at 37℃ for 2 h. The absorbance at 460 nm was measured using a microplate reader, and the cell proliferation rate was calculated.

### 5-Ethynyl-2’-deoxyuridine (EdU) staining

Cells were incubated with 50 µM EdU at 37℃ for 2 h, followed by elution with PBS buffer. After fixation with 4% PFA, cells were treated with glycine and permeabilized with 0.5% TritonX-100. They were then incubated in 100 µL 1 × Apollo staining solution for 0.5 h in the dark. For nuclear staining, cells were treated with 1 × Hoechst 33,342 for 0.5 h in the dark, and excess Hoechst was removed with PBS buffer. Fluorescence microscopy was used to observe and photograph cell fluorescence, with ImageJ software calculating the Apollo/Hoechst positive ratio.

### Colony formation assay

Transfected cells were plated at a density of 5 × 10^^2^ cells per well in 6-well plates and cultured at 37℃ for 14 days. Following fixation with 4% paraformaldehyde (PFA), cells were stained with 0.5% crystal violet for 30 min. Colonies were then visualized and imaged using a colony counter, and the number of colonies was quantified using ImageJ software.

### Cell titer Glo (CTG) assay

Cell viability was accessed by CTG assay according to the manufacturer’s protocol. Briefly, HCT116 and RKO cells were seeded in 96 well white culture plates at a density of 1000 cells/well in 200 µl of the cell culture medium. Cells were incubated at 37˚C, 5% CO_2_ for 24 h. Following incubation, 100 µl of media was added to each well and treated with 100 µl of CTG reagent and kept on a shaker for 2 min. The plates were kept at room temperature for 10 min to stabilize the luminescence signal. Luminescence was measured using microtiter plate ELISA reader (Bio Tek, Winooski, Vermont, USA).

### Luciferase reporter assay

Luciferase reporter plasmids containing the promoter sequences for CDKN1A and the targeting sequence of 6 mA/6 mA-Mut were transfected to measure transcriptional activation. Control groups received equivalent amounts of the pLG4 empty vector and processed identically to experimental transfection conditions. After 24 h, a Dual-Luciferase Reporter Assay System was employed to quantify firefly and renilla luciferase activities. Relative luciferase activity was calculated by normalizing firefly luciferase activity to renilla luciferase activity.

### Chromatin Immunoprecipitation (ChIP)-PCR

ChIP assays were conducted using kits from Beyotime. HCT116 cells (1 × 10^^7^) were crosslinked with 1% formaldehyde, lysed, and genomic DNA was sheared by sonication. Anti-6 mA or anti-p53 antibodies were used to precipitate DNA fragments corresponding to 6 mA peaks. Quantitative real-time PCR was performed to determine the fold-enrichment of each fragment, with input DNA serving as a control. Primer sequences are listed in Table S2.

### Stiffness measurement with nanoindentation

Tissue stiffness was measured using a ferrule-top nanoindenter setup with a PIUMA controller/drive, as previously described [25]. A probe with a 0.49 N/m spring constant and a 9 mm spherical indentation tip was used for the measurements. Specimens were fixed to slides, and the probe was brought into contact with the material surface to record load indentation and load-time data. Indentations were depth-controlled at 10 μm, with a loading and unloading period set to 2 s.

### Data acquisition

RNA expression microarray data (GSE150936 and GSE117632) were sourced from the Gene Expression Omnibus (GEO) database [27, 28]. Colon adenocarcinoma (COAD) and rectum adenocarcinoma (READ) cohorts were accessed from The Cancer Genome Atlas (TCGA) via the UCSC Xena repository. Immunohistochemical staining data for ALKBH1 in normal and CRC tissues were obtained from the Human Protein Atlas (HPA) [29]. Staining intensity was extracted from the “Antibody staining” section to quantitatively assess ALKBH1 protein expression levels.

### Statistical analysis

All measurements are shown as mean ± SEM from at least 3 independent biological experiments. Statistical analyses were performed using GraphPad Prism 9.0.0. Independent Student’s *t*-test was used to compare the difference between two groups. One-way ANOVA with Turkey’s multiple comparison was used to compare the difference among three or more groups. The 2-sided P-value < 0.05 was considered statistically significant.

## Results

### Decreased DNA 6 ma modifications are correlated with CRC stiffening

**Fig. 1:**
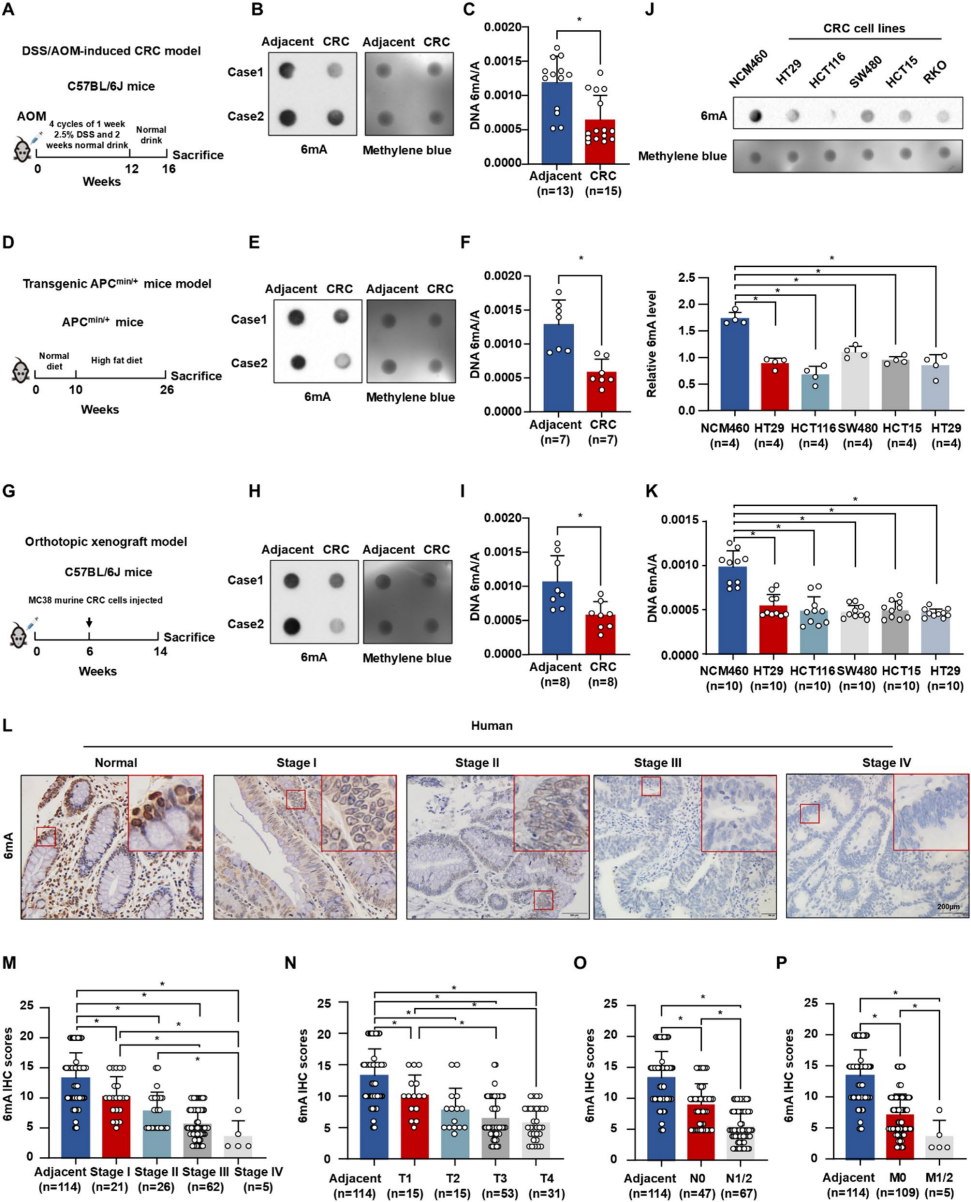
DNA 6 mA levels gradually decreased as CRC development. (**A**) Schematic diagram of DSS/AOM induced CRC model in C57BL/6J mice. (**B-C**) DNA 6 mA levels in DSS/AOM CRC model were determined by dot blot (**B**) and ELISA assays (**C**). (**D**) Schematic diagram of transgenic APC ^min/+^ mice induced CRC. (**E-F**) Dot blot (E) and ELISA assays (**F**) detected DNA 6 mA levels in transgenic APC ^min/+^ CRC model. (**G**) Schematic diagram of DNA 6 mA levels in orthotopic xenograft CRC model. (**H-I**) Dot blot (**H**) and ELISA assays (**I**) of DNA 6 mA levels derived from orthotopic xenograft CRC model. (**J**) The DNA 6 mA levels in the NCM460 cell line and CRC cell lines (HT29, HCT116, SW480, HCT15, and RKO) were determined by dot blot (up panel) followed by relative quantitative analysis (down panel). (**K**) ELISA assays indicated DNA 6 mA levels in NCM460 cell line and CRC cell lines (HT29, HCT116, SW480, HCT15, and RKO). (**L**) Representative IHC of 6 mA modification levels in human adjacent tissue and CRC tissues at different stages. (**M-P**) The histograms of 6 mA IHC scores at different AJCC stages (**M**), T stages(N), N stages (**O**), and M stages (**P**) Data are represented as mean ± SEM. *P < 0.05

To ascertain the aberrant regulation of DNA 6 mA modification in CRC, we quantified DNA 6 mA levels using dot blot and ELISA assays. Primary CRC tumors exhibited significantly reduced 6 mA levels compared to adjacent normal colorectal tissues in multiple models: DSS/AOM-induced CRC mice (Fig. 1A-C), transgenic APC^min/+^ mice (Fig. 1D-F), and an orthotopic xenograft model (Fig. 1G-I). Analysis of genomic DNA from CRC cell lines (HT29, HCT116, SW480, HCT15, RKO) versus normal colon epithelial NCM460 cells also revealed significantly reduced DNA 6 mA levels in CRC cells (Fig. 1J-K). Immunohistochemistry (IHC) confirmed markedly lower DNA 6 mA levels in primary human and mouse CRC tumors compared to adjacent normal tissues (Fig. 1L and Figure S1A-B). Staining intensity and area assessment indicated higher scores in tumor tissues (Figure S1C-E). Furthermore, during human CRC progression, patients with stage III/IV disease exhibited significantly decreased 6 mA levels compared to stage I patients (Fig. 1L-M). Integrating these findings with clinical data revealed a negative correlation between 6 mA levels and the Tumor-Node-Metastasis (TNM) stage of CRC (Fig. 1N-P). Additionally, primary orthotopic xenograft tumors with lung metastasis displayed significantly lower 6 mA levels than those without metastasis (Figure S1F-G). Collectively, these data demonstrate that reduced DNA 6 mA modification correlates with CRC progression.

**Fig. 2:**
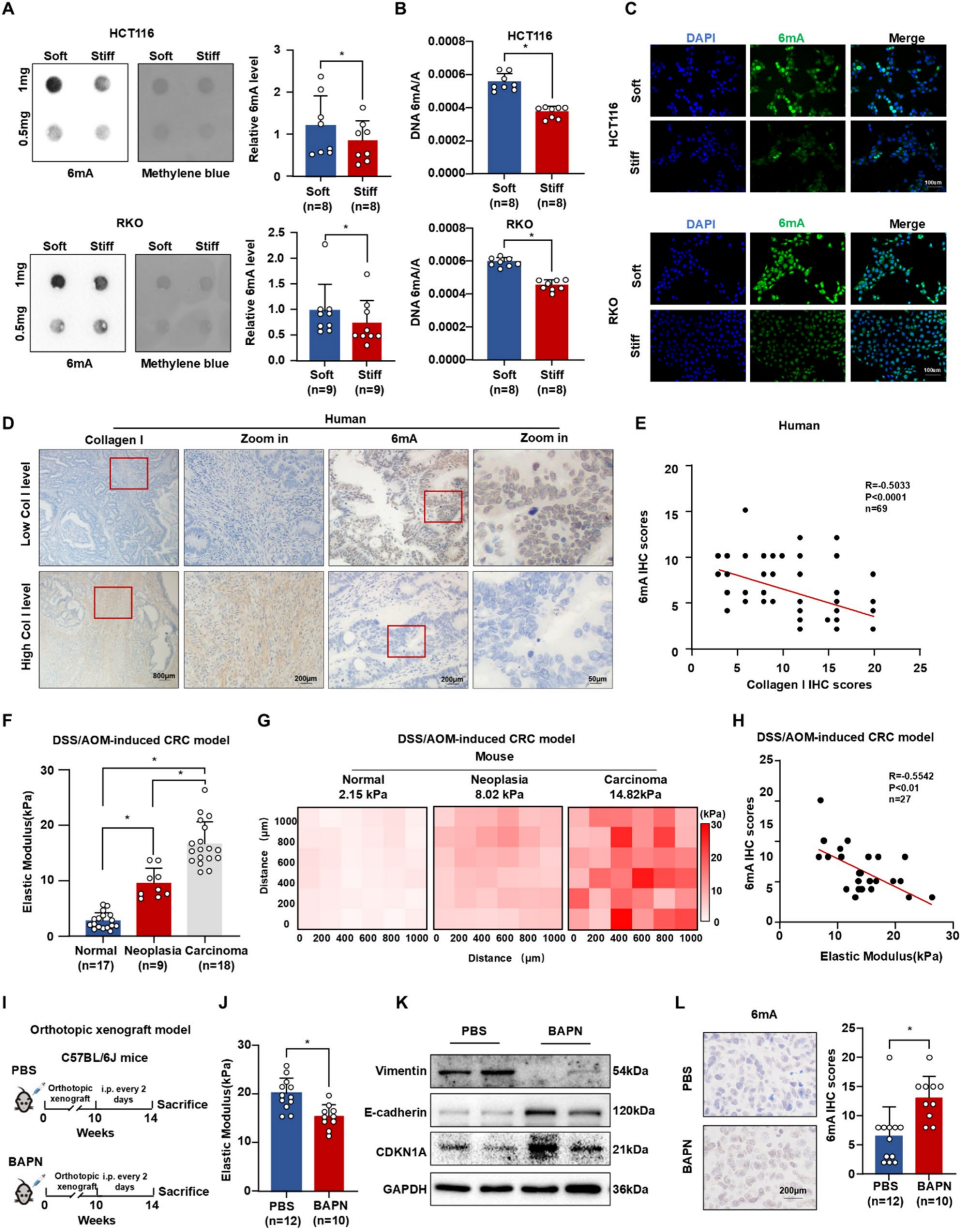
Extracellular matrix stiffness was associated with a reduction of 6 mA level. (**A**) Dot blot (left panel) and relative quantitative statistics (right panel) showed DNA 6 mA level of HCT116 cells (up panel) and RKO cells (down panel) stimulated by soft and stiff substrate. (**B-C**) ELISA assay (**B**) and IF staining (**C**) indicated DNA 6 mA levels of HCT116 cells (up panel) and RKO cells (down panel) stimulated by soft and stiff substrate. (**D-E**) Representative IHC (**D**) and Spearman’s correlation analysis (**E**) of collagen I and 6 mA in human CRC tissues. (**F-G**) Histogram (**F**) and heat map (**G**) of elastic modulus among normal colorectal tissues, AOM-induced neoplasia tissues, and AOM-induced CRC tissues. (**H**) Spearman’s correlation analysis of 6 mA IHC scores and elastic modulus in neoplasia tissues and CRC tissues of DSS/AOM-induced mice. (**I**) The BAPN treatment process for orthotopic xenograft mice model. (**J**) Histogram of elastic modulus in PBS-treated xenografts and BAPN-treated xenografts. (**K**) Western blot of vimentin, E-cadherin, CDKN1A in PBS-treated xenografts and BAPN-treated xenografts, using GAPDH as a control. (**L**) Representative IHC (left panel) and relative quantitative statistics (right panel) of 6 mA modification levels in PBS-treated xenografts and BAPN-treated xenografts Data are represented as mean ± SEM. *P < 0.05

To investigate how substrate stiffness influences DNA 6 mA modifications, CRC cells were cultured on polyacrylamide (PA) gels of varying stiffness. DNA 6 mA levels were downregulated in HCT116 and RKO cells under stiff substrate conditions (Fig. 2A-C). Given collagen’s preeminent role as the primary ECM scaffolding protein, forming parallel fiber arrays crucial for tensile resilience, we bolstered our observations by assessing DNA 6 mA levels in a collagen-analyzed patient cohort (*n* = 69). IHC revealed a significant negative correlation between 6 mA levels and collagen levels (*R* = -0.5033, *P* < 0.0001) (Fig. 2D-E). Nanoindentation analysis of the elastic modulus in mouse tissues showed increased stiffness during CRC progression, with average values of 2.74 kPa for normal colon tissues (*n* = 17), 9.508 kPa for colorectal neoplasia (*n* = 9), and 16.596 kPa for colorectal carcinoma tissues (*n* = 18) (Fig. 2F-G and Figure S2). DNA 6 mA modification levels were negatively correlated with the elastic modulus of primary CRC tissues (*n* = 27, *R* = -0.5542, *P* < 0.01) (Fig. 2H). To manipulate tissue stiffness, we treated DSS/AOM-induced CRC and orthotopic xenograft mouse models with β-aminopropionitrile (BAPN), a specific lysyl oxidase (LOX) inhibitor (Fig. 2I and Figure S3A). Nanoindentation confirmed significantly decreased stiffness in CRC tissues upon BAPN treatment (Fig. 2J and Figure S3B). BAPN treatment increased epithelial-mesenchymal transition (EMT) marker E-cadherin and tumor suppressor CDKN1A expression, while decreasing Vimentin expression (Fig. 2K). IHC demonstrated that matrix softening rescued DNA 6 mA levels in CRC tissues (Fig. 2L and Figure S3C). The correlation analysis indicated that the level of DNA 6 mA modification was negatively correlated with the elastic modulus (Figure S3D, *n* = 22, *R* = -0.8137, *P* < 0.0001). Taken together, these findings indicate that substrate stiffening decreases DNA 6 mA modifications and promotes tumor progression in CRC.

### Substrate stiffening promotes 6 ma demethylases ALKBH1 upregulation

**Fig. 3:**
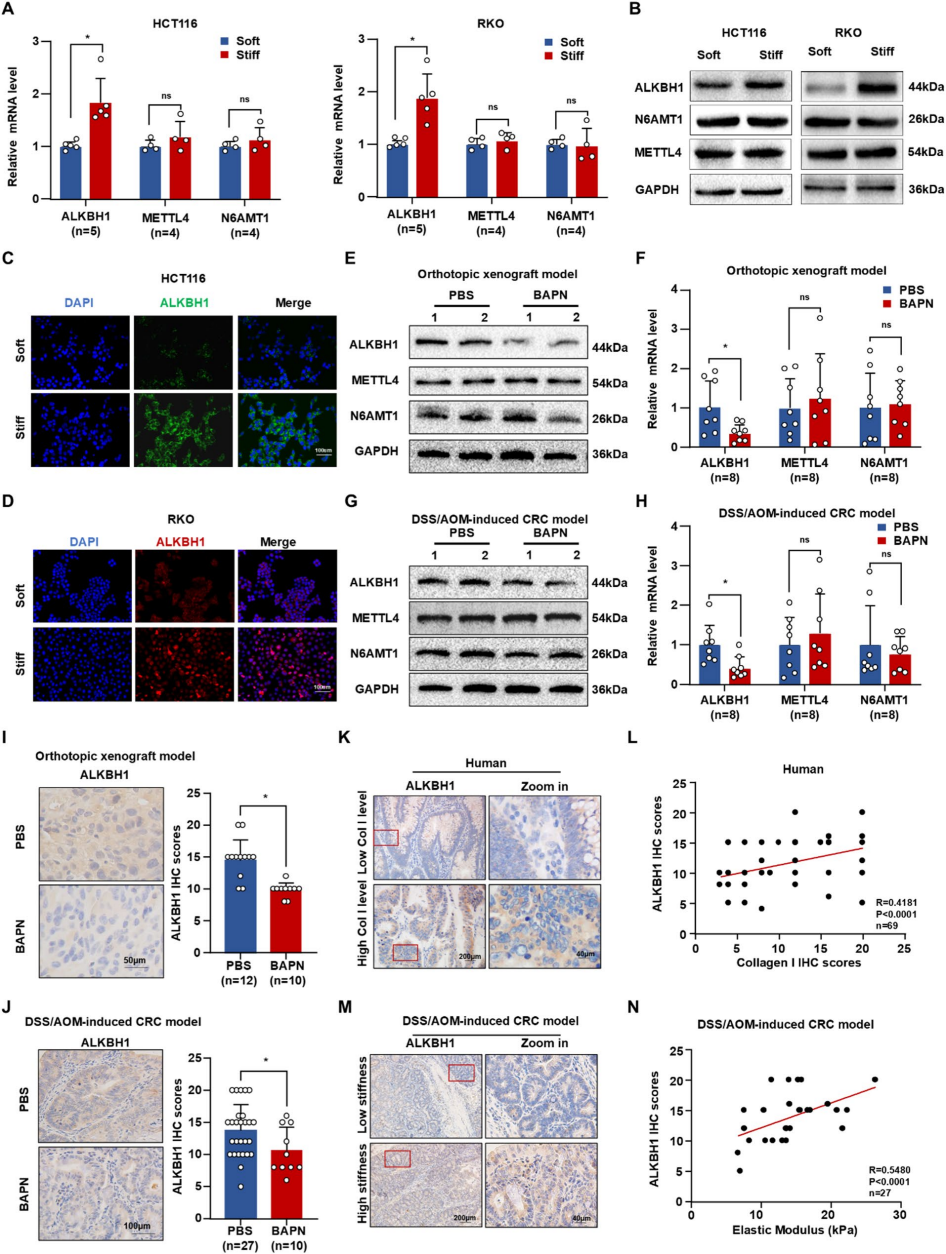
Substrate stiffness upregulates ALKBH1. (**A**) Histograms of mRNA expression of ALKBH1, METTL4 and N6AMT1 in HCT116 (left panel) and RKO cells (right panel) stimulated by soft and stiff substrate. (**B**) Western blot of ALKBH1, METTL4 and N6AMT1 in HCT116 (left panel) and RKO cells (right panel) stimulated by soft and stiff substrate, using GAPDH as a control. (**C-D**) IF staining indicated ALKBH1 levels of HCT116 cells (**C**) and RKO cells (**D**) stimulated by soft and stiff substrate. (**E-F**) Western blot (**E**) and qRT-PCR (F) of ALKBH1, METTL4 and N6AMT1 in orthotopic xenograft model treated by PBS and BAPN, using GAPDH as a control. (**G-H**) Western blot (**G**) and qRT-PCR (H) of ALKBH1, METTL4 and N6AMT1 in DSS/AOM-induced CRC model treated by PBS and BAPN, using GAPDH as a control. (**I-J**) Representative IHC (left panel) and relative quantitative statistics (right panel) of ALKBH1 in orthotopic xenograft model (**I**) and DSS/AOM-induced CRC model (**J**) treated by PBS and BAPN. (**K**) Representative IHC of ALKBH1 in low collagen I group and high collagen I group of human CRC tissues. (**L**) Spearman’s correlation analysis of ALKBH1 IHC scores and collagen I IHC scores in human CRC tissues. (**M**) Representative IHC of ALKBH1 in low stiffness group and high stiffness group of DSS/AOM induced CRC mice. (**N**) Spearman’s correlation analysis of ALKBH1 IHC scores and elastic modulus in AOM/DSS-induced CRC tissues Data are represented as mean ± SEM. *P < 0.05; ns, not significant

Demethylases and methyltransferases dynamically regulate DNA 6 mA methylation levels [18, 30]. To investigate how matrix stiffness modulates DNA 6 mA methylation, we evaluated the expression of the 6 mA demethylase ALKBH1 and the 6 mA methyltransferases N6AMT1 and METTL4. Our results demonstrated that ALKBH1 expression is transcriptionally upregulated in response to substrate stiffening, whereas N6AMT1 and METTL4 expression levels remained largely unchanged (Fig. 3A-D). Furthermore, ALKBH1 expression (but not METTL4 or N6AMT1) was significantly reduced following BAPN treatment, which softens the matrix, in both orthotopic xenograft (Fig. 3E-F) and DSS/AOM-induced CRC tissues (Fig. 3G-H). IHC staining confirmed suppression of ALKBH1 in the BAPN-treated groups (Fig. 3I-J and Figure S3E). We further assessed ALKBH1 expression levels in the human collagen-analyzed cohort (*n* = 69) by IHC, revealing a significant positive correlation between ALKBH1 expression and collagen levels (*R* = 0.4181, *P* < 0.0001) (Fig. 3K-L). Additionally, in the nanoindentation-analyzed mouse CRC cohort (*n* = 27), ALKBH1 expression assessed by IHC (Fig. 3M) showed a positive correlation with tissue stiffness (*R* = 0.5480, *P* < 0.0001) (Fig. 3N). Collectively, these findings indicate that matrix stiffness positively regulates ALKBH1 expression.

### Matrix stiffness regulated the transcription of 6 ma-modified genes via ALKBH1-mediated 6 ma demethylation

**Fig. 4:**
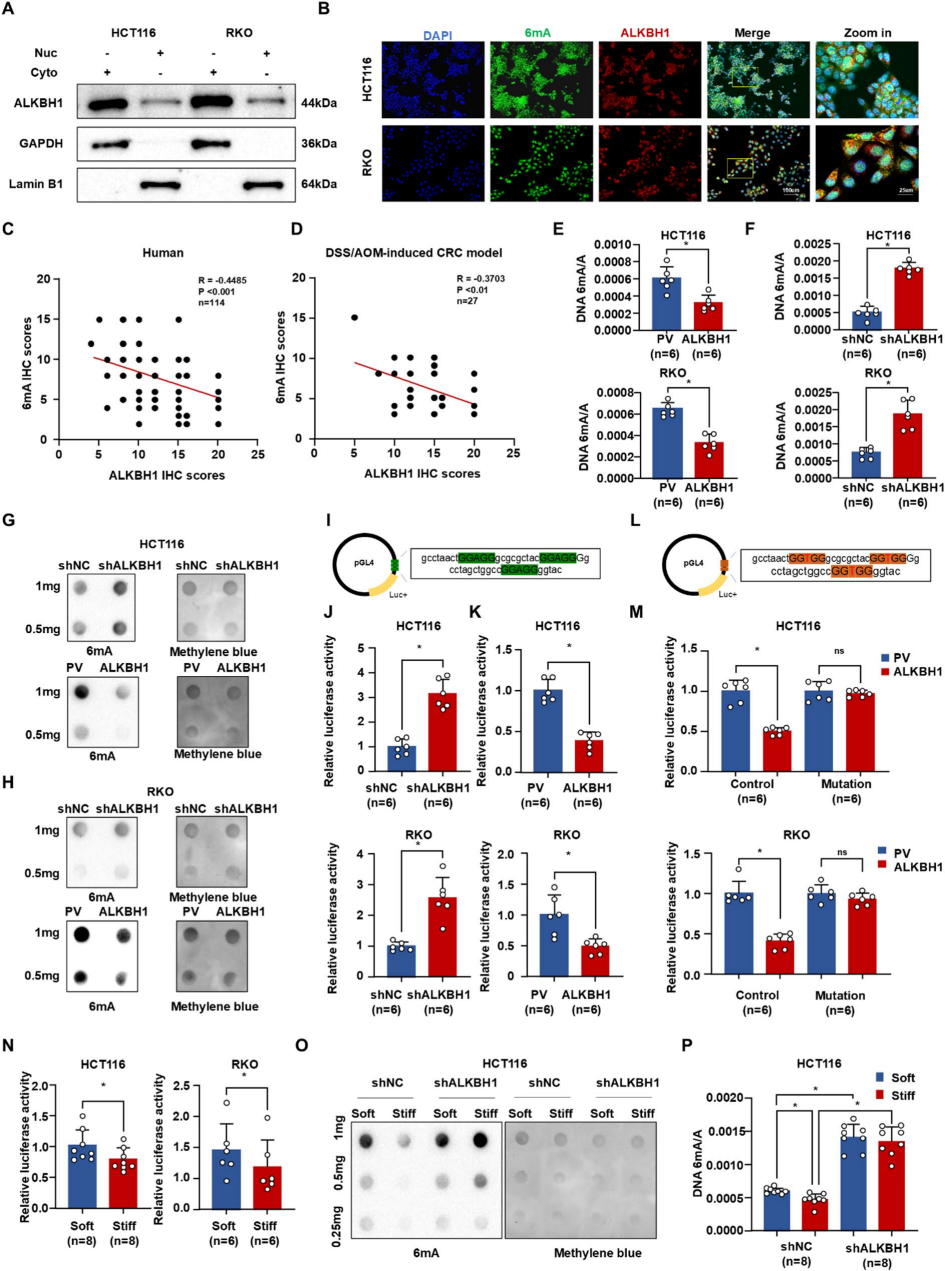
Demethylase ALKBH1 was responsible for 6 mA downregulation stimulated by stiffness. (**A**) Western blot of ALKBH1 distribution in HCT116 and RKO cells, using GAPDH as a cytoplasmic control and Lamin B1 as a nuclear control. (**B**) Representative confocal microscopy images of ALKBH1 and 6 mA in HCT116 and RKO cells. (**C-D**) Spearman’s correlation analysis of 6 mA and ALKBH1 IHC scores in human CRC (**C**) and AOM/DSS-induced neoplasia and CRC tissues (**D**). (**E-F**) Elisa assays of 6 mA levels in HCT116 (up panel) and RKO cells (down panel) transfecting ALKBH1 plasmids (**E**) or shALKBH1 plasmids (**F**). (**G-H**) Dot blot of 6 mA levels in HCT116 (**G**) and RKO cells (**H**) transfecting shALKBH1 plasmids (up panel) or ALKBH1 plasmids (down panel). (**I**) Diagram of 6 mA PGL4 luciferase reporter containing 3 X GGAGG motif. (**J-K**) Histograms of luciferase activity of 6 mA in HCT116 (up panel) and RKO (down panel) with ALKBH1 downregulation (**J**) and ALKBH1 overexpression (**K**). (**L**) Diagram of mutant 6 mA PGL4 luciferase reporter containing 3 X GGTGG motif. (**M**) Histograms of luciferase activity of 6 mA and mutant 6 mA in HCT116 (up panel) and RKO (down panel) transfecting ALKBH1 plasmid and plasmid vector. (**N**) Histograms of 6 mA luciferase activity of HCT116 (left panel) and RKO cells (right panel) stimulated by soft and stiff substrate. (**O-P**) Dot blot (**O**) and ELISA (**P**) showed DNA 6 mA levels of HCT116 cells with knockdown ALKBH1 and control after soft and stiff substrate stimulation. Data are represented as mean ± SEM. *P < 0.05; ns, not significant; Nuc: nucleus; Cyto: cytoplasm

We investigated the association between ALKBH1 expression and DNA 6 mA modifications in CRC. Nuclear-cytoplasmic fractionation revealed that ALKBH1 is predominantly localized in both the cytoplasm and nucleus of HCT116 and RKO cells (Fig. 4A), consistent with prior reports [18, 28]. Confocal microscopy further demonstrated co-localization of ALKBH1 protein with 6 mA immunostaining in these compartments (Fig. 4B). Notably, ALKBH1 expression exhibited a significant negative correlation with DNA 6 mA modification levels in primary CRC tumors (human cohort: *n* = 69, *R*=-0.4485, *P* < 0.001; mouse cohort: *n* = 27, *R*=-0.3703, *P* < 0.01) (Fig. 4C-D). Dot blot and ELISA analyses confirmed that ALKBH1 negatively regulates global DNA 6 mA levels in CRC cells (Fig. 4E-H; Figure S4).

To determine whether ALKBH1-mediated 6 mA demethylation modulates transcriptional activity, we cloned a 3X GGAGG motif into a PGL4 luciferase reporter vector (Fig. 4I). Ectopic ALKBH1 expression significantly suppressed luciferase activity in HCT116 and RKO cells, whereas ALKBH1 knockdown enhanced it (Fig. 4J-K). In contrast, ALKBH1 manipulation had no effect on a control reporter containing mutated motifs (Fig. 4L-M). We next examined whether matrix stiffness regulates DNA 6 mA through ALKBH1. Luciferase assays under varying substrate stiffness showed that matrix stiffening increased reporter activity (Fig. 4N). Critically, ALKBH1 knockdown prior to stiffening reversed the stiffness-induced reduction in DNA 6 mA levels (Fig. 4O-P). These results demonstrate that matrix stiffness modulates transcriptional activity of 6 mA-modified genes via ALKBH1-dependent demethylation.

### Expression pattern and clinical relevance of ALKBH1 in CRC tissues

**Fig. 5:**
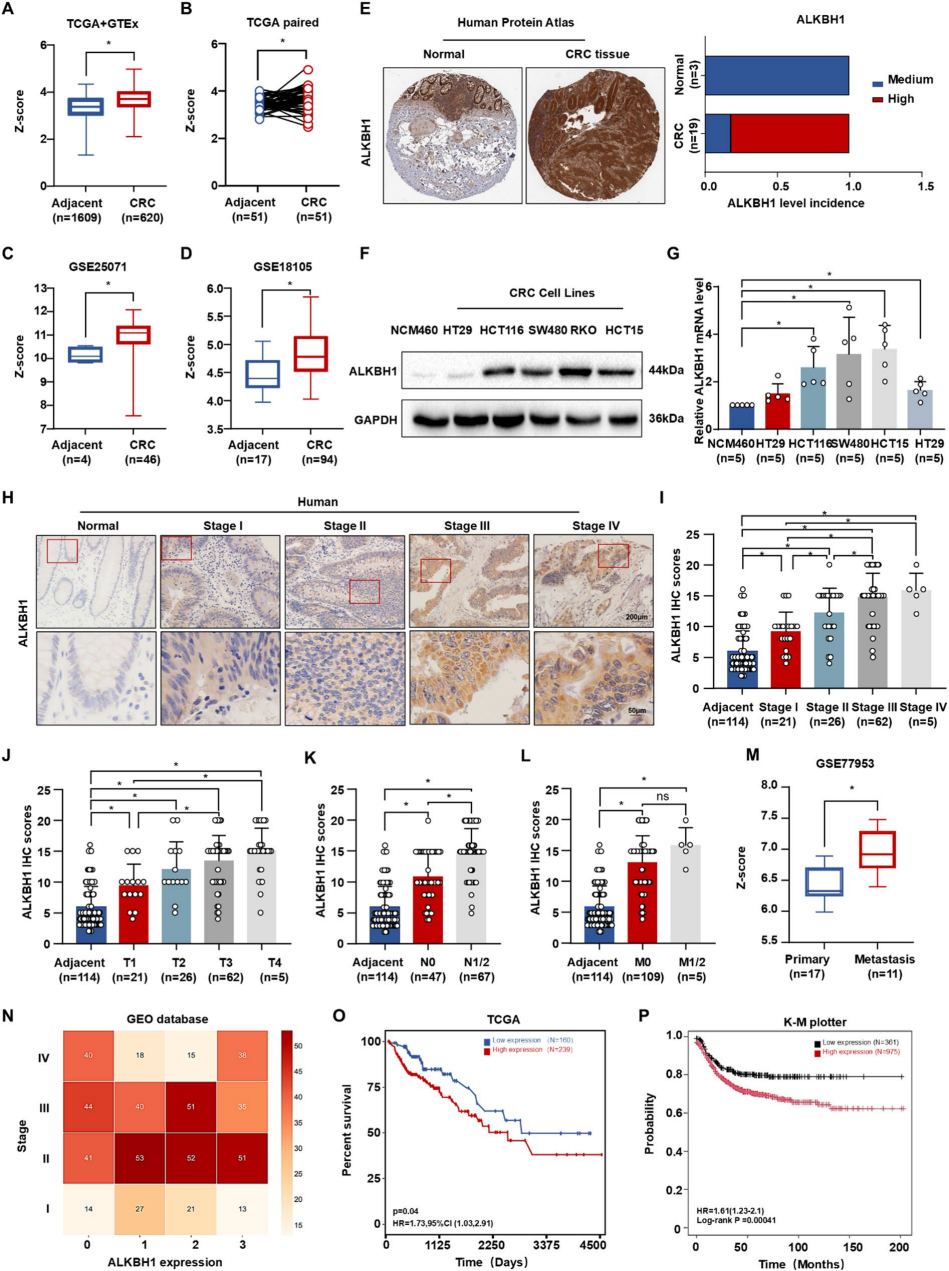
Demethylase ALKBH1 was associated with poor prognosis in CRC. (**A**) Boxplot presented ALKBH1 expression in CRC and adjacent normal tissues using the TCGA combined with GTEx datasets. (**B**) Scatter plot of ALKBH1 expression using TCGA paired datasets. (**C-D**) Boxplot presented ALKBH1 expression in CRC and adjacent normal tissues from GSE25071 (**C**) and GSE18105 datasets (**D**). (**E**) Representative IHC (left panel) and IHC (right panel) analysis of ALKBH1 protein expression in normal tissues and CRC tissues from HPA immunostaining dataset. (**F-G**) The protein (**F**) and mRNA levels (**G**) of ALKBH1 in CRC cells lines (HT29, HCT116, SW480, RKO and HCT15) and normal colon endothelial cell line (NCM460), using GAPDH as a control. (**H**) Representative IHC of ALKBH1 in human adjacent normal tissues and CRC tissues under different stages. (**I-L**) The histograms of ALKBH1 scores at different AJCC stages (**I**), T stages(**J**), N stages (**K**), and M stages (**L**). (**M**) Boxplot presented ALKBH1 expression in primary CRC tissues and metastasis tissues from GSE77953 dataset. (**N**) Heat map of ALKBH1 expression in CRC tissues of different stages from TCGA, GSE20970, GSE77955, GSE103512, GSE128449, GSE156451, and GSE211831 datasets. (**O-P**) Kaplan-Meier plots of overall survival of CRC patients from TCGA (**O**) and Kaplan-Meier Plotter datasets (**P**), stratified according to mean ALKBH1 expression. Data are represented as mean ± SEM. *P < 0.05; ns, not significant

To delineate the expression profile of ALKBH1 during CRC progression, we first assessed its clinical significance using multi-omics datasets. Analysis of TCGA-GTEx revealed significant ALKBH1 upregulation in CRC tumors versus adjacent normal tissues at the mRNA level (Fig. 5A-B), corroborated by GEO datasets (GSE25071, GSE18105) (Fig. 5C-D). Consistently, the Human Protein Atlas (HPA) public immunostaining dataset demonstrated elevated ALKBH1 protein expression in primary CRC tissues (Fig. 5E). In vitro, CRC cell lines exhibited elevated ALKBH1 expression compared to normal colon NCM460 cells (Fig. 5F-G). In vivo, western blot and IHC analyses of DSS/AOM-induced CRC tissues confirmed significant ALKBH1 upregulation (Figure S5A-B). Our cohort-based IHC assays further revealed increased ALKBH1 in CRC versus adjacent normal tissues (Fig. 5H and Figure S5C). Notably, ALKBH1 expression correlated significantly with advanced T and N stages (*P* < 0.05), but not M stage (Fig. 5I-L), while GEO dataset analysis (GSE77953) demonstrated elevated ALKBH1 transcripts in metastases relative to primary tumors (Fig. 5M). By integrating clinical staging data from TCGA and six GEO datasets (GSE20970, GSE77955, GSE103512, GSE128449, GSE156451, GSE211831), we stratified patients into stage I-IV cohorts. ALKBH1 expression levels were categorized into quartiles (0–3) using the interquartile range method, revealing a positive correlation with advancing clinical stage (Fig. 5N). Critically, Kaplan-Meier analysis demonstrated significantly prolonged median overall survival in patients with low versus high ALKBH1 expression (Fig. 5O-P). Collectively, these findings establish ALKBH1 as a consistently upregulated driver of CRC malignancy and a potential prognostic biomarker.

### ALKBH1 upregulation promotes CRC cell proliferation

To delineate ALKBH1’s role in matrix-stiffness-driven proliferation, we manipulated its expression in CRC cells (Figure S6). ALKBH1 overexpression enhanced proliferation (Figure S7A-C), while knockdown significantly suppressed it (Figure S7D-F). For the in vivo assessment of ALKBH1’s tumorigenic effects, subcutaneous xenografts of ALKBH1-stable-knockdown HCT116 cells in nude mice was conducted. ALKBH1 overexpression accelerated tumor growth by analyzing of excised tumor photos (Figure S8A), 28-day tumor growth curves (*P* < 0.001, Figure S8B), and tumor weights (*P* < 0.001, Figure S8C). The immunohistochemical results indicated that the expression of ALKBH1 was significantly decreased in the in orthotopic xenograft tumor tissues of the shALKBH1 group, and the corresponding degree of DNA 6 mA modification increased. Consistently, proliferation markers PCNA and Ki67 were downregulated in shALKBH1 tumors (Figure S8D-E). These findings establish ALKBH1 as critical for CRC tumorigenesis.

### The demethylase activity of ALKBH1 is required for CRC cell proliferation

**Fig. 6:**
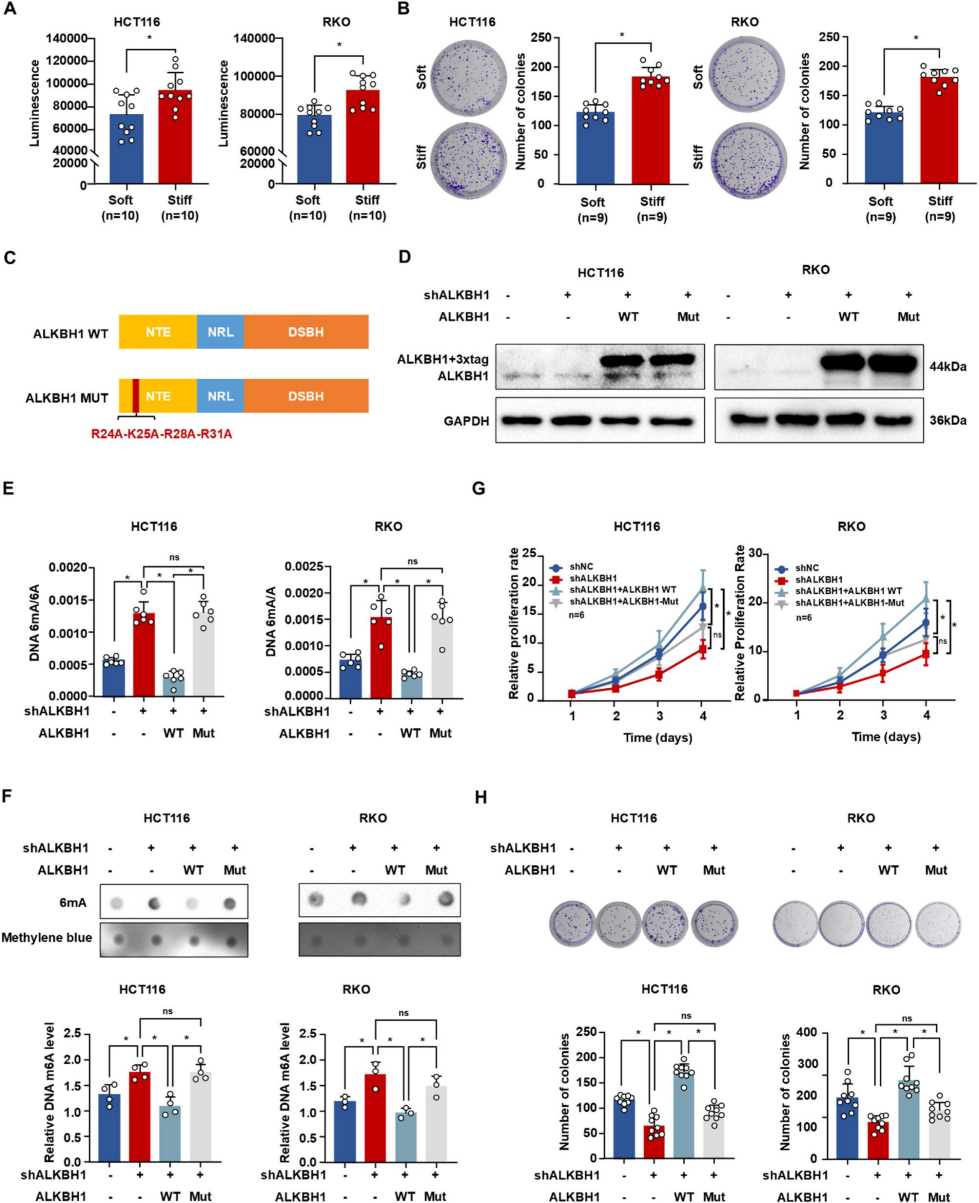
The demethylase activity of ALKBH1 promoted CRC proliferation. (**A**) Histogram of CTG luminescence in HCT116 (left panel) and RKO cells (right panel) stimulated by soft and stiff substrate. (**B**) Colony assay and quantitative analysis of HCT116 (left panel) and RKO cells (right panel) stimulated by soft and stiff substrate. (**C**) Mutation sites of ALKBH1 in the demethylation activity domain. (**D**) Western blot of ALKBH1 expression in HCT116 (left panel) and RKO cells (right panel) overexpressing wild-type or mutant ALKBH1 plasmids after shRNA-targeting ALKBH1 transfection, using GAPDH as a control. (**E**) Histograms of 6 mA ELISA assay in HCT116 (left panel, *n* = 6 per group) and RKO cells (right panel, *n* = 6 per group) overexpressing wild-type or mutant ALKBH1 plasmids after shALKBH1 transfection. (**F**) Dot blots (up panel) and relative quantitative analysis (down panel) of 6 mA levels in HCT116 (left panel, *n* = 4 per group) and RKO cells (right panel, *n* = 3 per group) overexpressing wild-type or mutant ALKBH1 plasmids after shRNA-targeting ALKBH1 transfection. (**G**) CCK8 assays of HCT116 (left panel) and RKO cells (right panel) transfecting wild-type or mutant ALKBH1 plasmids after shALKBH1 transfection. (**H**) Colony assays (up panel) and quantitative statistics (down panel) of HCT116 (left panel, *n* = 9 per group) and RKO cells (right panel, *n* = 9 per group) transfecting wild-type or mutant ALKBH1 plasmids after shALKBH1 transfection Data are represented as mean ± SEM. *P < 0.05; ns, not significant; WT, wild-type; Mut, mutant

To further elucidate the functional contribution of ALKBH1 in matrix stiffness-induced CRC cell proliferation, cells were cultured on substrates of varying stiffness. Quantitative assessment using CTG luminescent viability assays and colony formation assays demonstrated that substrate stiffening significantly enhanced the proliferative capacity of CRC cells (Fig. 6A-B). We subsequently investigated whether the DNA 6 mA demethylase activity of ALKBH1 is mechanistically required for regulating this proliferative response. To specifically inhibit ALKBH1’s catalytic function toward genomic DNA 6 mA modifications, we engineered a quadruple-alanine substitution mutant (R24A/K25A/R28A/R31A) targeting evolutionarily conserved residues within the substrate-binding pocket that are essential for demethylase activity (Fig. 6C) [25]. Rescue experiments were performed by reconstituting ALKBH1-knockdown cells with either wild-type (WT) or mutant ALKBH1 expression constructs (Fig. 6D). Restoration of WT ALKBH1 expression substantially reversed the DNA 6 mA hypermethylation phenotype induced by ALKBH1 depletion, whereas the catalytic mutant failed to restore 6 mA demethylation (Fig. 6E-F). Correspondingly, functional rescue of proliferation deficits and clonogenic impairment was exclusively observed in cells expressing WT ALKBH1, with no restorative effects conferred by the enzymatically defective mutant (Fig. 6G-H). These collective findings confirm that the DNA 6 mA demethylase activity of ALKBH1 is crucially required for promoting stiffness-driven CRC cell proliferation.

### ALKBH1 facilitated CRC cells proliferation by attenuating CDKN1A transcription

**Fig. 7:**
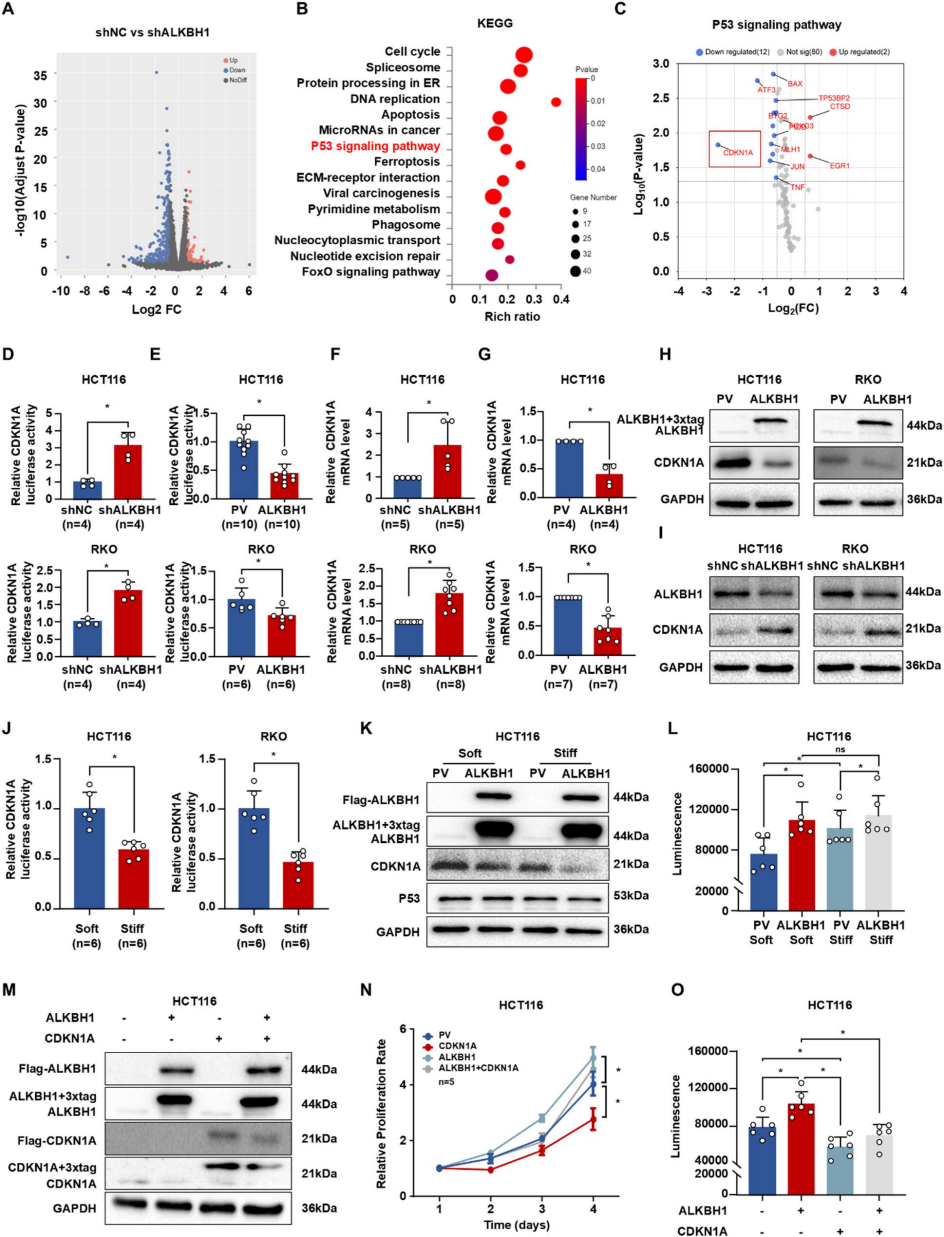
ALKBH1 inhibited the transcription of CDKN1A. (**A-B**) Volcano plot (**A**) and KEGG-enrichment pathways (**B**) of differentially expressed genes between shALKBH1 HCT116 cells and controls. (**C**) Volcano plot of genes related to P53 pathway RNA microarray in HCT116 cells between shALKBH1 and controls. (**D-E**) Histograms of luciferase activity of CDKN1A in HCT116 (up panel) and RKO cells (down panel) with ALKBH1 knockdown (**D**) and ALKBH1 expression (**E**). (**F-G**) Histograms of CDKN1A mRNA expression in HCT116 (up panel) and RKO cells (down panel) with ALKBH1 knockdown (**F**) and ALKBH1 expression (**G**). (**H-I**) Western blot of CDKN1A and ALKBH1 expression in HCT116 (left panel) and RKO cells (right panel) with ALKBH1 overexpression (**H**) and ALKBH1 knockdown (**I**), using GAPDH as a control. (**J**) Histograms of CDKN1A luciferase activity in HCT116 (left panel) and RKO cells (right panel) stimulated by soft and stiff substrate. (**K**) Western blot showed Flag, ALKBH1, CDKN1A and P53 expression in HCT116 cells with ALKBH1 overexpression and control after soft and stiff substrate stimulation, using GAPDH as a control. (**L**) Histogram of CTG luminescence in in HCT116 cells with ALKBH1 overexpression and control after soft and stiff substrate stimulation, *n* = 6 per group. (**M**) Western blot of Flag, ALKBH1 and CDKN1A expression in HCT116 cells transfecting ALKBH1 and/or CDKN1A plasmids, using GAPDH as a control. (N-O) CCK8 assays (*n* = 5 per group) (**N**) and CTG luminescence histogram (*n* = 6 per group) (O) of HCT116 cells transfecting ALKBH1 and/or CDKN1A plasmids. Data are represented as mean ± SEM. *P < 0.05; ns, not significant

To elucidate the mechanism by which matrix stiffness-induced ALKBH1 promotes CRC progression, we performed RNA sequencing in HCT116 cells. Applying an adjusted P-value threshold of 0.05 and |log2FC| ≥1, we identified 817 upregulated and 343 downregulated genes in ALKBH1-deficient cells (Fig. 7A). Notably, CRC-promoting oncogenes were significantly downregulated while tumor-suppressive genes were upregulated (Figure S9). KEGG pathway analysis revealed enrichment of differentially expressed genes (DEGs) in cell cycle regulation, apoptosis, and p53 signaling pathways (Fig. 7B). Validation using GEO datasets (GSE150936: ALKBH1-deficient human diploid cells; GSE117632: glioblastoma cells) confirmed overlapping DEGs similarly enriched in proliferation, cell cycle, and p53 pathways (Figure S10A-C). Given the critical role of the P53 signaling pathway in CRC cell proliferation, we focused on its mediation of ALKBH1 effects. RNA microarray analysis of p53 pathway components (Fig. 7C) and luciferase reporter assays (Fig. 7D-E) demonstrated that CDKN1A (p21), a key p53 target, is transcriptionally repressed by ALKBH1. This finding was corroborated by GSE150936 and GSE117632 datasets (Figure S10D). qRT-PCR (Fig. 7F-G) and western blotting (Fig. 7H-I) confirmed ALKBH1-mediated suppression of CDKN1A at mRNA and protein levels. Matrix stiffening inhibited CDKN1A expression (Fig. 7J), an effect counteracted by ALKBH1 overexpression (Fig. 7K). Importantly, substrate stiffening potentiated the pro-proliferative effect of ALKBH1 overexpression (Fig. 7L). Rescue experiments demonstrated that exogenous CDKN1A expression partially attenuated ALKBH1-driven proliferation (Fig. 7M-O). Collectively, these results establish that ALKBH1 promotes CRC proliferation through transcriptional downregulation of CDKN1A.

### ALKBH1 attenuates the DNA binding capacity of P53 to CDKN1A promoter region

**Fig. 8:**
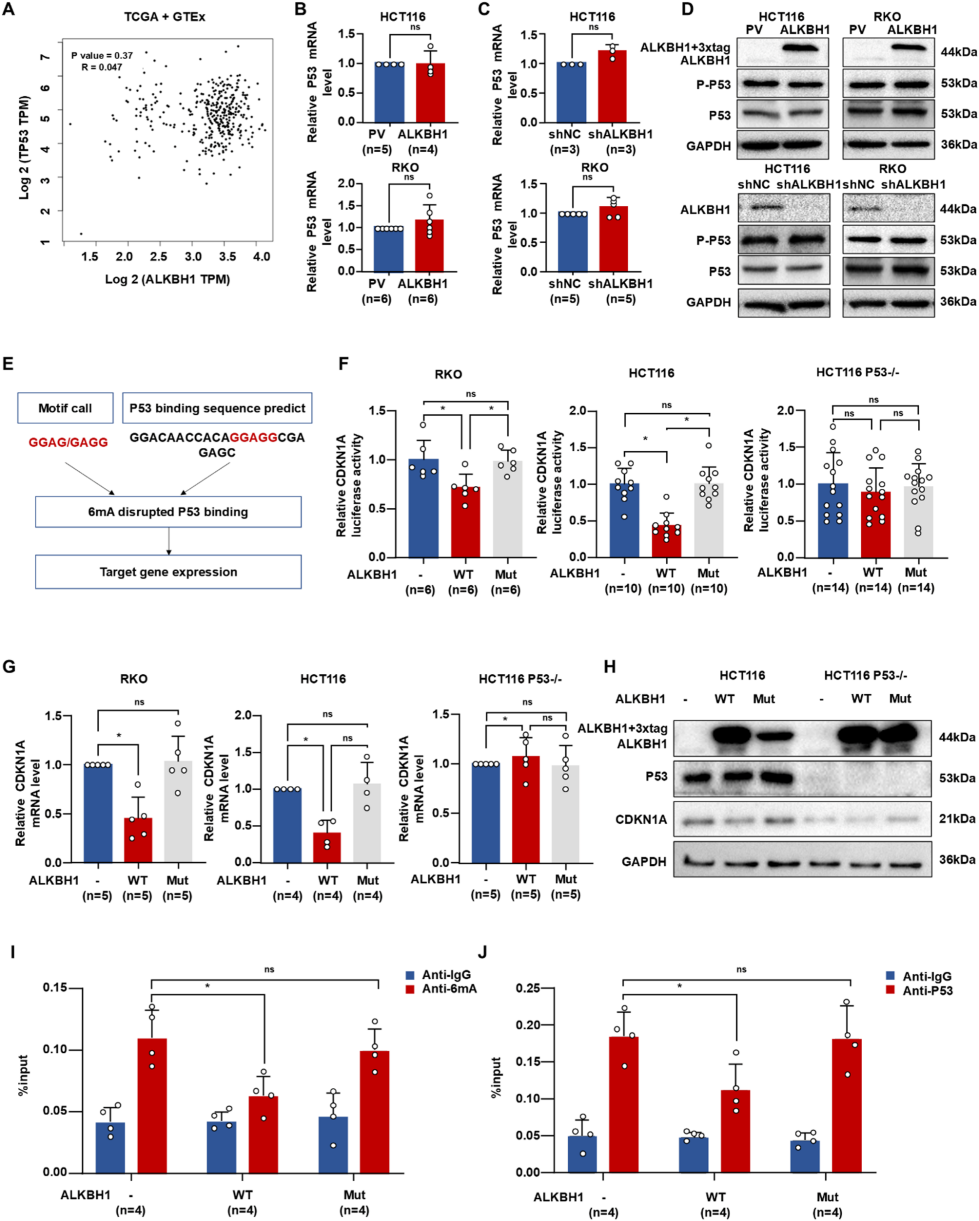
ALKBH1 attenuates P53 binding to the p21 promoter. (**A**) Spearman correlation analysis of TP53 and ALKBH1 from TCGA combing with GTEx database. (**B-C**) Histograms of P53 mRNA expression in HCT116 (up panel) and RKO cells (down panel) with ALKBH1 overexpression (**B**) and ALKBH1 knockdown (**C**). (**D**) Western blot showed ALKBH1, P-P53, and P53 expression in HCT116 (left panel) and RKO cells (right panel) with ALKBH1 overexpression (up panel) and ALKBH1 knockdown (down panel), using GAPDH as a control. (**E**) Predictive motif of p53-bound methylation-modified regions. (**F-G**) Histograms of CDKN1A luciferase activity (**F**) and CDKN1A mRNA expression (**G**) in RKO (left panel), HCT116 (middle panel), and p53-knockout HCT116 cells (right panel) overexpressing vector, wild-type ALKBH1, and mutant ALKBH1 plasmids. (**H**) Western blot of ALKBH1, P53, and CDKN1A in HCT116 and p53-knockout HCT116 cells overexpressing control, wild-type ALKBH1, and mutant ALKBH1 plasmids, using GAPDH as a control. (I) ChIP assay detected 6 mA modification levels on the CDKN1A promoter using anti-6 mA or control IgG antibody, combined with qPCR assay in HCT116 cells transfected with plasmid vector, wild-type ALKBH1 and mutant ALKBH1 plasmids. (J) ChIP assay showed binding ability of p53 to the CDKN1A promoter using anti-p53 or control IgG antibody, combined with qPCR assay in HCT116 cells transfected with plasmid vector, wild-type ALKBH1 and mutant ALKBH1 plasmids Data are represented as mean ± SEM. *P < 0.05; ns, not significant; WT, wild-type; Mut, mutant

To investigate the mechanism by which ALKBH1 regulates CDKN1A expression, we first assessed the correlation between ALKBH1 and P53 using GEPIA. This analysis revealed no significant direct association (*R* = 0.047, *P* = 0.37; Fig. 8A). Consistent with this, neither P53 expression nor activity was affected by ALKBH1 in CRC cells (Fig. 8B-D). Given previous reports identifying P53 as a potential transcription factor for DNA sequences harboring 6 mA modifications [15], we hypothesized that ALKBH1-induced 6 mA demethylation primarily targets P53-mediated transcription (Fig. 8E). To validate this hypothesis, we generated a P53-deleted HCT116 cell line. In both HCT116 and RKO cells, overexpression of ALKBH1-WT, but not ALKBH1-Mut, significantly inhibited CDKN1A promoter activity and mRNA levels (Fig. 8F-G). Crucially, P53 deletion abolished the inhibitory effect of ALKBH1-WT on both CDKN1A expression and luciferase reporter activity (Fig. 8F-H). Further analysis demonstrated that ALKBH1-WT overexpression, but not ALKBH1-Mut, reduced 6 mA enrichment specifically within the CDKN1A promoter region, confirming ALKBH1-mediated 6 mA demethylation at this locus (Fig. 8I). Correspondingly, ALKBH1-WT overexpression suppressed P53 binding to the CDKN1A promoter, an effect not observed with ALKBH1-Mut overexpression (Fig. 8J). Collectively, these results indicate that ALKBH1 suppresses CDKN1A mRNA expression in CRC cells, at least in part, by inhibiting P53-mediated transcription through promoter 6 mA demethylation.

## Discussion

ECM stiffness is an essential solid tumor microenvironment for tumor progression and metastasis [4], it plays an essential role in facilitating tumor development. Previous study reported that dysadherin/MMP9 axis enhances ECM proteolytic activity and activates cancer-associated fibroblast remodeling matrix stiffness, leading to CRC progression [31]. Our present study delves deeper into this realm, uncovering a critical pathway through which matrix stiffness influences DNA 6 mA modification levels to foster CRC proliferation, supported by both in vivo and in vitro experimental evidence.

The mechanism we uncovered reveals that ECM stiffness upregulates the expression of the demethylase ALKBH1. This leads to a reduction in the DNA 6 mA methylation level of CDKN1A, a tumor suppressor gene. The decreased methylation hinders the binding of the transcription factor P53 to the CDKN1A promoter region, thereby inhibiting CDKN1A expression and promoting CRC progression. In CRC tissues, the global reduction of 6 mA signifies widespread dysregulation of the DNA methylation machinery. However, the localized demethylation at CDKN1A promoters introduces complexity to the epigenetic regulation of CRC. ECM stiffness-induced ALKBH1 upregulation selectively targets tumor-suppressive genes, driving CRC progression. While the global reduction of 6 mA establishes a permissive epigenetic landscape, the localized ALKBH1-mediated demethylation executes precise oncogenic programming. This study sheds light on how tumor biomechanical signals can alter epigenetic modifications, and how the epigenetic modification levels of tumor suppressor genes can regulate tumor progression. It also suggests that diagnosing stiffness in CRC may offer predictive power for CRC prognosis, and that softening matrix stiffness and targeting the key demethylase ALKBH1 could hold therapeutic potential.

Previous studies have illustrated that matrix stiffness can promote CRC progression and metastasis by upregulating tumor-related genes such as HSF4, activating lipolysis, and enhancing angiogenesis [8, 9]. Moreover, the increase in tumor ECM stiffness is a crucial factor contributing to the poor sensitivity of tumor chemotherapy. Softening ECM stiffness may thus be a potential therapeutic strategy to enhance drug sensitivity and improve prognosis [32, 33]. Our findings align with these prior reports, providing substantial in vivo and in vitro evidence that stiffness promotes colorectal cancer progression, and that inhibiting CRC proliferation can be achieved by softening matrix hardness using BAPN.

Matrix stiffness has been documented to regulate the expression of key tumor genes through various signaling pathways, including the integrin β1/Piezo1 activation/Ca^2+^ influx pathway, RhoA/ROCK pathway, YAP-POSTN-integrin mechanotransduction signaling, and YAP/TEAD4/ACADL axis [34–37]. Regarding the epigenetics of substrate stiffness regulation, ECM stiffness has been shown to reversibly promote DNA hypomethylation in the YAP promoter region, thereby increasing YAP expression and nuclear translocation in gastric cancer [38]. Importantly, our study demonstrates that matrix stiffness can regulate the DNA 6 mA level, indicating that biomechanical signals can influence gene expression through epigenetic mechanisms. It also suggests that DNA 6 mA demethylation modifications of tumor suppressor genes may play a decisive role in CRC development.

To explore the DNA 6 mA downregulation in CRC tissues caused by matrix stiffness, we investigated three key methylation-modifying enzymes: ALKBH1, METTL4, and N6AMT1 [30, 39]. Our results indicate that ALKBH1 is the key demethylase involved in epigenetic modifications mediated by mechanical signal transduction. Furthermore, we found that ALKBH1 decreases the promoter’s 6 mA level of CDKN1A, thereby inhibiting the transcriptional expression of this tumor suppressor gene and exacerbating CRC. In human glioblastoma, ALKBH1 has been reported to induce 6 mA demethylation, silencing tumor suppressor genes and activating downstream hypoxic response elements [28]. It has also been shown to enhance the expression of DDX18 by removing DNA 6 mA modifications and regulating promoter activity, thereby promoting the proliferation of head and neck squamous cell carcinoma [20]. Consistent with these previous studies, ALKBH1 is identified as an oncogene that regulates the P53-CDKN1A pathway, offering a novel therapeutic target for inhibiting CRC proliferation.

Given the significant role of ALKBH1 in tumor development, the development of ALKBH1 inhibitors has garnered attention. Xiong et al. synthesized the small molecule inhibitors of ALKBH1 (termed 13 h and 16) for glioblastoma, which can regulate cellular 6 mA levels [40]. Subsequently, Li et al. reported that 1 H-pyrazole-4-carboxylic acid derivative 29 is a highly effective ALKBH1 inhibitor, and they developed a prodrug 29 (named 29E) [41].Treatment with 29E significantly inhibited gastric cancer cell viability by increasing 6 mA abundance, and the hydrolysate of 29 exhibited high exposure in mice after administration [41]. While the development of ALKBH1 inhibitors has shown promising results for cancer treatment, several critical issues remain to be addressed for their clinical application, such as determining the minimum effective drug concentration, conducting safety and toxicity evaluations, and assessing efficacy in organoids.

Looking ahead, it is worthwhile to explore the potential of ALKBH1 inhibitors and targeting matrix stiffness in cancer therapy. Although our study bridges the gap between matrix stiffness and DNA methylation modifications in CRC, certain limitations need to be acknowledged and addressed. Firstly, while we observed that ECM stiffness activates the transcriptional expression of ALKBH1, the precise mechanistic signaling underlying this regulation requires further elucidation. A more detailed understanding of how biomechanical signals are transduced into biological signals to modulate ALKBH1 expression is essential. Secondly, the development of molecular compounds that can specifically inhibit ALKBH1 would greatly enhance the therapeutic potential for CRC treatment. Thirdly, although our study demonstrates a strong association between ALKBH1 activation and CRC proliferation in vivo and in vitro, in vivo experiments using ALKBH1 knockout and p53 knockout cells are necessary to establish causal relationships. Lastly, while we have explored the stiffness-ALKBH1-6 mA mechanism in CRC, a genome-wide analysis would be valuable to determine its universality across various cancers, potentially paving the way for pan-cancer treatments. Despite these limitations, our study provides valuable insights into the role of matrix stiffness in CRC progression and highlights ALKBH1 as a promising therapeutic target. Future research should focus on addressing these limitations to further advance our understanding and improve therapeutic strategies for CRC.

## Conclusion

In conclusion, our study has demonstrated that ECM stiffness reduces the level of DNA 6 mA methylation through the action of the demethylase ALKBH1. Our exploration of the underlying molecular mechanisms has revealed that ALKBH1 diminishes the 6 mA level within the promoter region of CDKN1A. Subsequently, this reduction disrupts the interaction between the transcription factor P53 and the CDKN1A promoter, thereby inhibiting the activation of CDKN1A. Taken together, these findings indicate that extracellular matrix stiffness promotes CRC progression via an epigenetic modification involving DNA 6 mA methylation. These results not only provide novel insights into the role of ECM stiffness in CRC development but also offer a potential diagnostic marker and therapeutic target for patients with advanced CRC.

## Supplementary Information

Below is the link to the electronic supplementary material.

Supplementary Material 1: Supplementary table and supplementary figure legend

Supplementary Material 2: Supplementary figures

## Data Availability

No datasets were generated or analysed during the current study.

## Abbreviations

6mA: N^6^-methyladenine
ALKBH1: AlkB homolog 1
AOM: azoxymethane
CDKN1A: cyclin dependent kinase inhibitor 1 A
ChIP: Chromatin immunoprecipitation
COAD: colonic adenocarcinoma
CRC: colorectal cancer
CTG: Cell Titer Glo
DSS: dextran sodium sulfate
ECM: extracellular matrix
EdU: 5-Ethynyl-2’-deoxyuridine
FBS: fetal bovine serum
IF: immunofluorescence staining
IHC: immunohistochemistry
i.p.: intraperitoneal
METTL4: methyltransferase 4
N6AMT1: N-6 adenine-specific DNA methyltransferase 1
OD: optical density
PA: polyacrylamide
PBS: phosphate buffer saline
PFA: paraformaldehyde
PV: plasmid vectors
qRT-PCR: real-time quantitative polymerase chain reaction
READ: rectal adenocarcinoma
SD: standard deviation
