# Supplementary material for: Extracellular matrix stiffness reduces DNA 6 ma level to facilitate colorectal cancer progression via disrupting P53 binding to CDKN1A promoter
Source: Exp Hematol Oncol. 2025 Aug 27;14:111. doi: 10.1186/s40164-025-00704-w (PMC12382035; doi:10.1186/s40164-025-00704-w)
Supplement: Supplementary file 1 — Supplementary Material 1: Supplementary table and supplementary figure legend [file 40164_2025_704_MOESM1_ESM.docx]

Supplementary table 1. List of clinical information of human CRC tissues

| **NO.** | **T stage** | **N stage** | **M stage** | **AJCC clinical stage (NO.7)** | **Sex** | **Age** |
| --- | --- | --- | --- | --- | --- | --- |
| 1 | T1 | N0 | M0 | 1 | female | 41 |
| 2 | T1 | N0 | M0 | 1 | male | 59 |
| 3 | T1 | N0 | M0 | 1 | female | 69 |
| 4 | T1 | N0 | M0 | 1 | female | 63 |
| 5 | T1 | N0 | M0 | 1 | male | 68 |
| 6 | T1 | N0 | M0 | 1 | female | 70 |
| 7 | T1 | N0 | M0 | 1 | male | 73 |
| 8 | T1 | N0 | M0 | 1 | male | 61 |
| 9 | T1 | N0 | M0 | 1 | male | 62 |
| 10 | T1 | N0 | M0 | 1 | male | 61 |
| 11 | T1 | N0 | M0 | 1 | male | 32 |
| 12 | T1b | N0 | M0 | 1 | female | 71 |
| 13 | T1b | N0 | M0 | 1 | male | 72 |
| 14 | T2 | N0 | M0 | 1 | female | 49 |
| 15 | T2 | N0 | M0 | 1 | female | 38 |
| 16 | T2 | N0 | M0 | 1 | female | 59 |
| 17 | T3 | N0 | M0 | 2 | male | 68 |
| 18 | T3 | N0 | M0 | 2 | female | 66 |
| 19 | T3 | N0 | M0 | 2 | male | 74 |
| 20 | T3 | N0 | M0 | 2 | male | 72 |
| 21 | T2 | N1 | M0 | 3 | female | 69 |
| 22 | T2 | N1a | M0 | 3 | female | 66 |
| 23 | T2 | N2a | M0 | 3 | male | 75 |
| 24 | T3 | N1 | M0 | 3 | female | 37 |
| 25 | T3 | N1 | M0 | 3 | male | 70 |
| 26 | T3 | N1 | M0 | 3 | female | 48 |
| 27 | T3 | N1 | M0 | 3 | female | 77 |
| 28 | T3 | N1 | M0 | 3 | female | 69 |
| 29 | T3 | N1 | M0 | 3 | female | 48 |
| 30 | T3 | N1 | M0 | 3 | male | 61 |
| 31 | T3 | N1b | M0 | 3 | male | 53 |
| 32 | T3 | N2a | M0 | 3 | male | 73 |
| 33 | T3 | N2a | M0 | 3 | male | 75 |
| 34 | T3 | N2b | M0 | 3 | male | 61 |
| 35 | T3 | N2b | M0 | 3 | female | 65 |
| 36 | T4 | N1 | M0 | 3 | female | 65 |
| 37 | T4 | N1 | M0 | 3 | female | 66 |
| 38 | T4 | N1 | M0 | 3 | male | 72 |
| 39 | T4 | N1 | M0 | 3 | female | 78 |
| 40 | T4 | N2 | M0 | 3 | male | 69 |
| 41 | T4a | N1 | M0 | 3 | female | 65 |
| 42 | T4a | N2a | M0 | 3 | male | 61 |
| 43 | T4a | N2a | M0 | 3 | female | 54 |
| 44 | T4a | N2b | M0 | 3 | male | 68 |
| 45 | T4b | N1 | M0 | 3 | male | 72 |
| 46 | T4b | N2a | M0 | 3 | male | 66 |
| 47 | T4a | N2 | M1 | 4 | female | 78 |
| 48 | T4b | N2 | M1 | 4 | male | 51 |
| 49 | T4b | N1 | M1a | 4 | female | 74 |

Supplementary table 2. List of primer sequences used for qRT–PCR

| Primer name | Sequence 5’-3’ |
| --- | --- |
| ALKBH1 FWD | AGAAGCGACTAAACGGAGACC |
| ALKBH1 REV | GGGAAAGGTGTGTAATGATCTGC |
| N6AMT1 FWD | GCAGGGGAGAACTTCGCTAC |
| N6AMT1 REV | CAGCGCGTTCAAAAGCAGAAA |
| METTL4 FWD | GGGAAGAGGCTACTTTGTCCT |
| METTL4 REV | GCCAAAGGGGGTTAAAACCTG |
| P53 FWD | GAGGTTGGCTCTGACTGTACC |
| P53 REV | TCCGTCCCAGTAGATTACCAC |
| P21 FWD | ACCATGTGGACCTGTCACTGT |
| P21 REV | TTAGGGCTTCCTCTTGGAGAA |
| GAPDH FWD | GGAGCGAGATCCCTCCAAAAT |
| GAPDH REV | GGCTGTTGTCATACTTCTCATGG |
| ChIP primer-1 FWD | CTGACTGCCCCTATTTGGGA |
| ChIP primer-1 REV | TGGAAAGCCCAAGCCTGAAG |
| ChIP primer-2 FWD | AGGGCTGCCTCTGCTCAATAA |
| ChIP primer-2 REV | AGTCCCAAATAGGGGCAGTC |
| ChIP primer-3 FWD | AGGCGTAAGCCACCACG |
| ChIP primer-3 REV | TGGATTTATTGTTTCTGACAAGTGG |
| ChIP primer-4 FWD | GGACTGGGCACTCTTGTCC |
| ChIP primer-4 REV | GACGGCCAGAAAGCCAATCA |
| ChIP primer-5 FWD | GACGGCCAGAAAGCCAATCA |
| ChIP primer-5 REV | GGTGAAAGGTGGAAAGCCCA |

**Supplementary Figure Legends**

**Supplementary Figure 1. The 6mA levels decreased in CRC and metastatic tissues**

(A-B) Representative IHC of 6mA modification levels in adjacent normal tissues and CRC tissues of DSS/AOM induced model (A) and Transgenic APC^min/+^ mice model (B). (C-E) Histogram of 6mA IHC scores in DSS/AOM induced model (C) and Transgenic APC^min/+^ mice model (D) and human adjacent normal tissues and CRC tissues (E). (F-G) Representative IHC (F) and relative quantitative statistics (G) of 6mA modification levels in lung tissues without and with metastatic cancer.

Data are represented as mean ± SEM. **P* < 0.05.

**Supplementary Figure 2.** **AOM/DSS induced CRC model**

Representative HE staining of neoplasia tissues and CRC tissues in AOM/DSS induced CRC mice.

**Supplementary Figure 3. BAPN increased DNA 6mA level**

(A) The BAPN treatment process in DSS/AOM induced CRC model. (B) Histogram of elastic modulus in PBS-treated and BAPN-treated DSS/AOM induced CRC model. (C) Representative IHC (left panel) and relative quantitative statistics (right panel) of 6mA levels in PBS-treated and BAPN-treated DSS/AOM induced CRC model. (D) Spearman’s correlation analysis of 6mA IHC scores and elastic modulus in CRC tissues of Orthotopic xenograft model. (E) Spearman’s correlation analysis of ALKBH1 scores and elastic modulus in CRC tissues of Orthotopic xenograft model.

Data are represented as mean ± SEM. **P* < 0.05.

**Supplementary Figure 4. ALKBH1 decreased DNA 6mA level**

(A-B) Relative quantitative statistics showed DNA 6mA level of HCT116 cells (A) and RKO cells (B) transfecting shALKBH1 plasmids (left panel) or wild-type ALKBH1 plasmids (right panel).

Data are represented as mean ± SEM. **P* < 0.05.

**Supplementary Figure 5. ALKBH1 elevated in CRC tissues**

(A-B) Representative IHC (A) and relative quantitative statistics (B) of ALKBH1 in DSS/AOM-induced mice. (C) Histogram of ALKBH1 IHC scores in human adjacent normal tissues and CRC tissues.

Data are represented as mean ± SEM. **P* < 0.05.

**Supplementary Figure 6. Overexpression and knockdown efficiency of ALKBH1**

(A-B) Western blot (A) and qRT-PCR (B) of ALKBH1 in HCT116 (left panel) and RKO cells (right panel) transfecting plasmid vector or ALKBH1 plasmids, using GAPDH as a control. (C-D) Western blot (C) and qRT-PCR (D) of ALKBH1 in HCT116 (left panel) and RKO cells (right panel) transfecting shNC, shALKBH1-1, or shALKBH1-2, using GAPDH as a control.

Data are represented as mean ± SEM. **P* < 0.05.

**Supplementary Figure 7. ALKBH1 promoted CRC proliferation in vitro**

(A) Line charts showed the proliferation rate of HCT116 (up panel) and RKO cells (down panel) with ALKBH1 knockdown and control from quantitative live-cell imaging. (B) Colony assay (left panel) and quantitative analysis (right panel) of HCT116 (up panel) and RKO cells (down panel) with ALKBH1 knockdown and control. (C) EdU assay (left panel) and semi-quantitative analysis (right panel) of HCT116 (up panel) and RKO cells (down panel) with ALKBH1 knockdown and control. (D) Line charts showed the proliferation rate of HCT116 (up panel) and RKO cells (down panel) transfecting plasmid vector or ALKBH1 plasmids from quantitative live-cell imaging. (E) Colony assay (left panel) and quantitative analysis (right panel) of HCT116 (up panel) and RKO cells (down panel) transfecting plasmid vector or ALKBH1 plasmids. (F) EdU assay (left panel) and semi-quantitative analysis (right panel) of HCT116 (up panel) and RKO cells (down panel) transfecting plasmid vector or ALKBH1 plasmids.

Data are represented as mean ± SEM. **P* < 0.05.

**Supplementary Figure 8. ALKBH1 promotes CRC proliferation in vivo**

(A) Representative imagines of xenograft tumors injected with HCT116 cells transfecting control or ALKBH1 knockdown (B) Line chart showed the proliferation rate of xenograft tumors after ALKBH1 knockdown or control. (C) Histograms of xenograft tumor weight after ALKBH1 knockdown or control. (D) Representative IHC of 6mA, ALKBH1, PCNA and Ki67 in xenograft tumor tissues after ALKBH1 knockdown or control. (E) Western blot of ALKBH1, P53 and CDKN1A expression of xenograft tumors after ALKBH1 knockdown or control, using GAPDH as a control.

Data are represented as mean ± SEM. **P* < 0.05.

**Supplementary Figure 9. ALKBH1 promoted tumor-related gene expression**

Heat map of differentially expressed genes from transcriptional profiling of HCT116 cells transfected with shNC and shALKBH1.

**Supplementary Figure 10. The p53 signaling pathway plays a crucial role in CRC proliferation**

(A) Venn gram of differentially expressed genes in GSE150936 and GSE117632 datasets. (B-C) GO pathway enrichment analysis (B) and KEGG pathway (C) of differentially differentiated genes from GSE150936 and GSE117632 datasets. (D) Genes expression related to P53 signaling pathway in GSE150936 and GSE117632 datasets.
