# Supplementary figures and images for: Extracellular matrix stiffness reduces DNA 6 ma level to facilitate colorectal cancer progression via disrupting P53 binding to CDKN1A promoter
Source: Exp Hematol Oncol. 2025 Aug 27;14:111. doi: 10.1186/s40164-025-00704-w (PMC12382035; doi:10.1186/s40164-025-00704-w)

Fig. S1

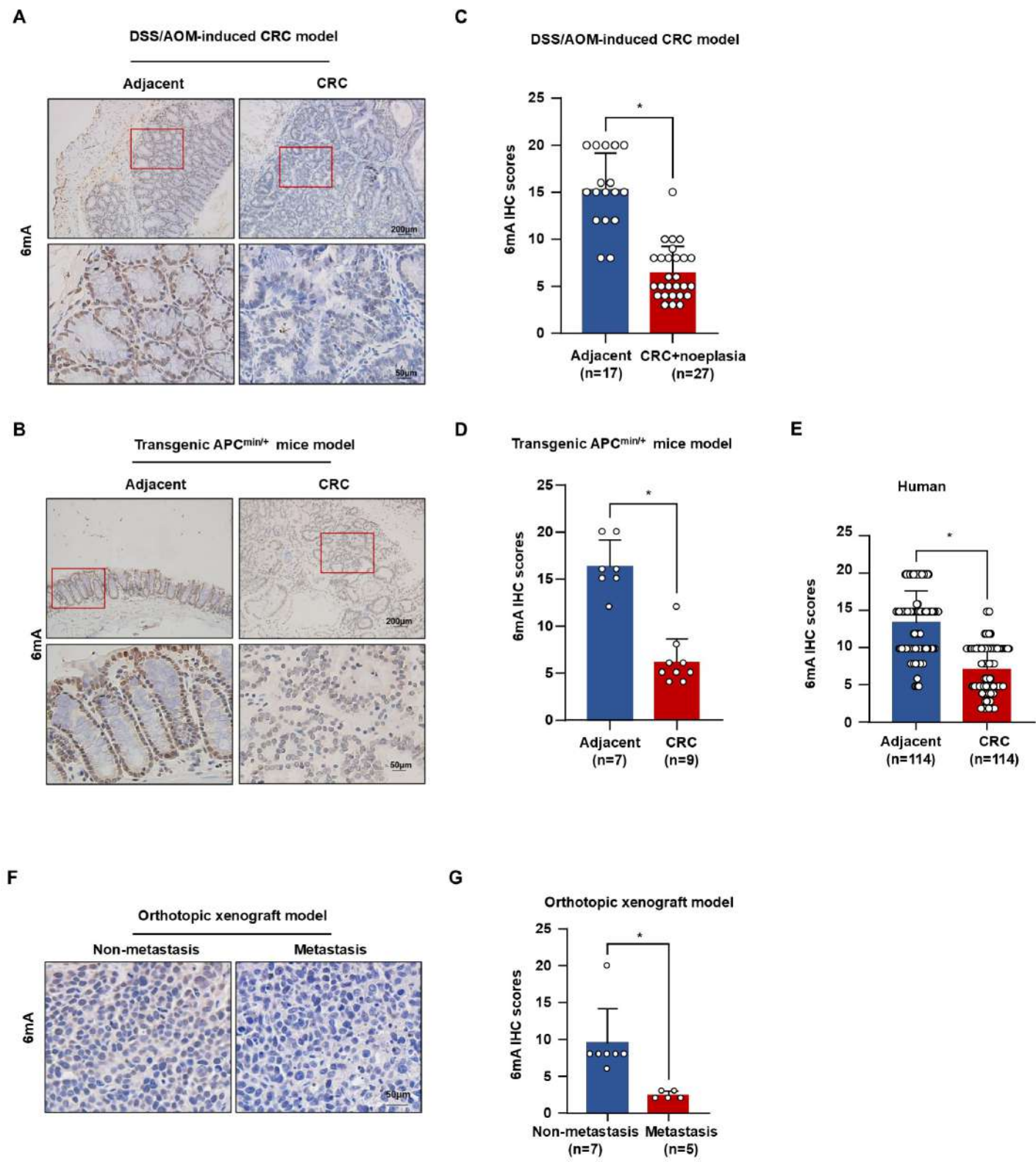

**Fig. S2**

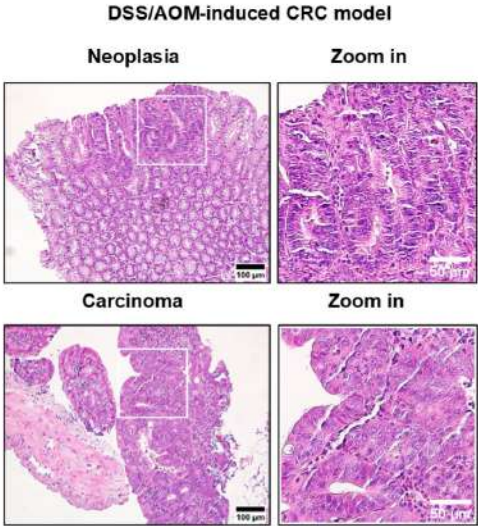

Fig. S3

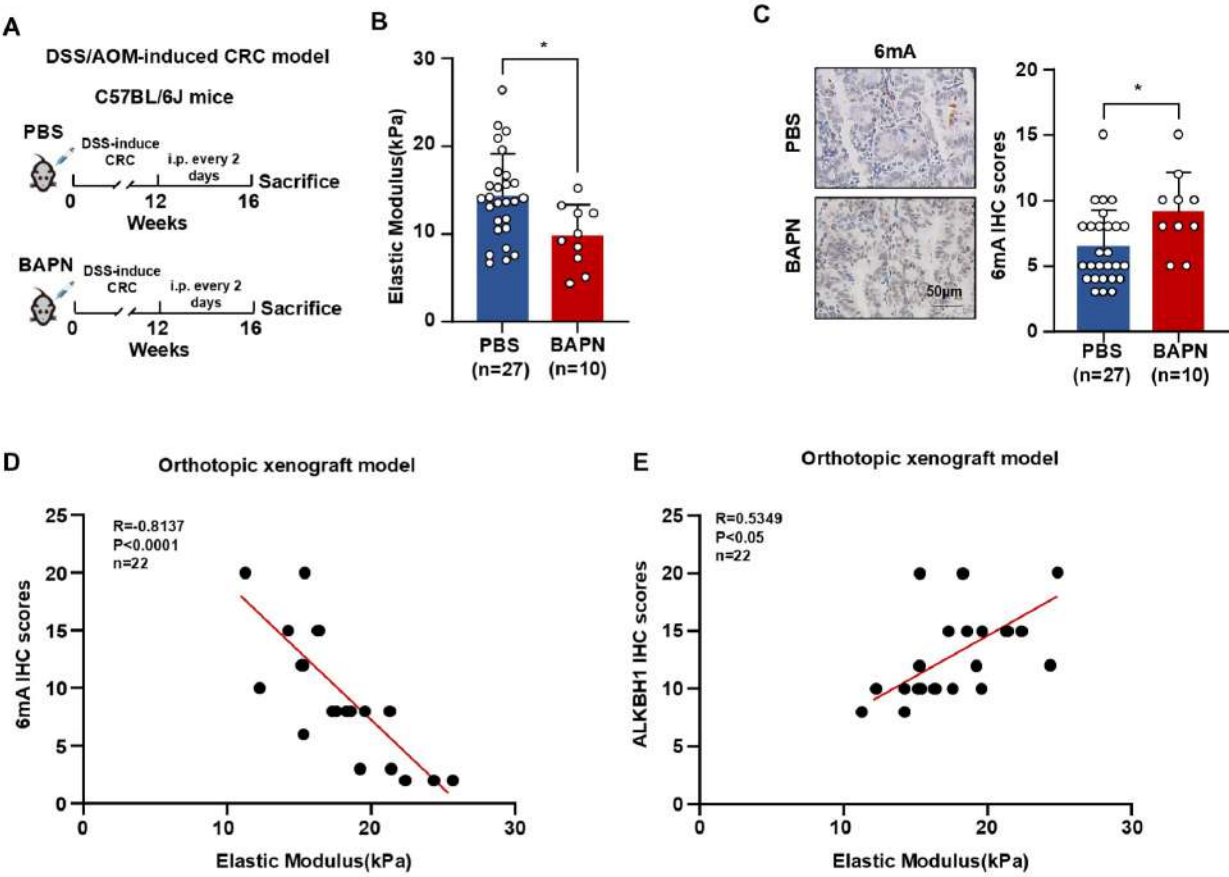

Fig. S4

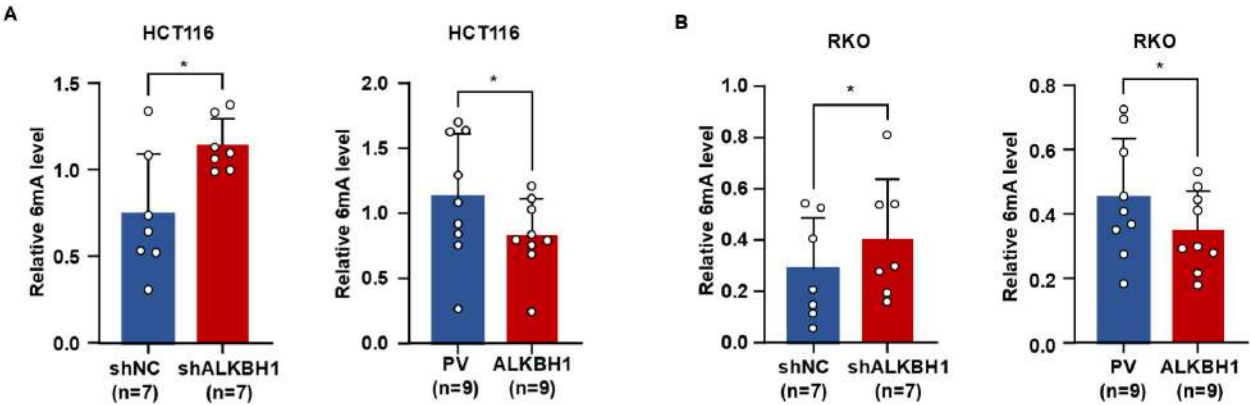

Fig. S5

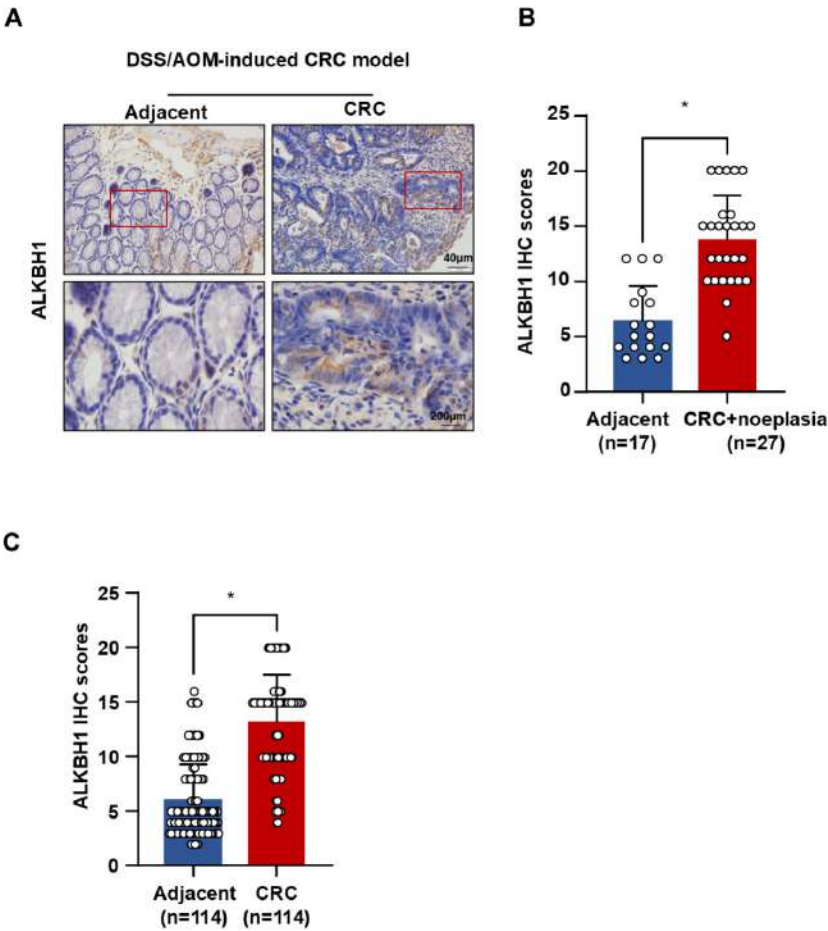

Fig. S6

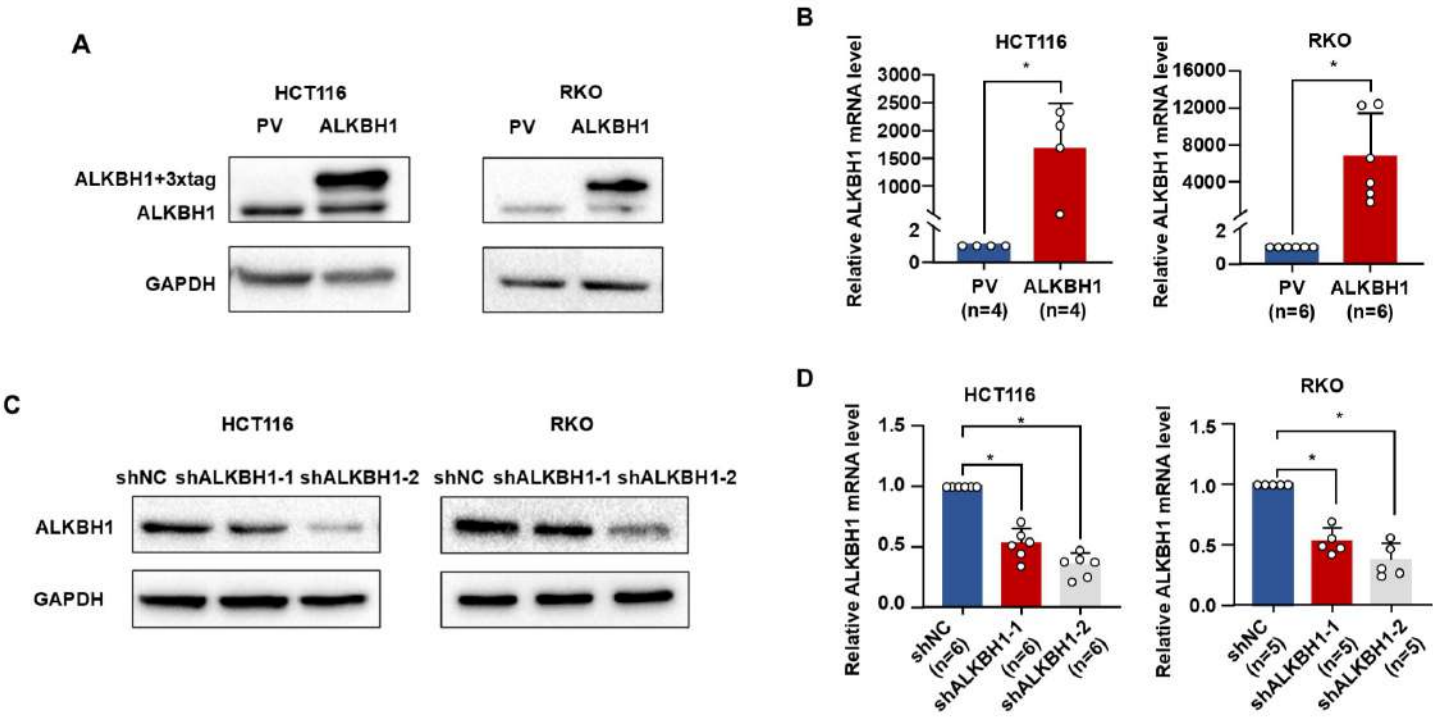

Fig. S7

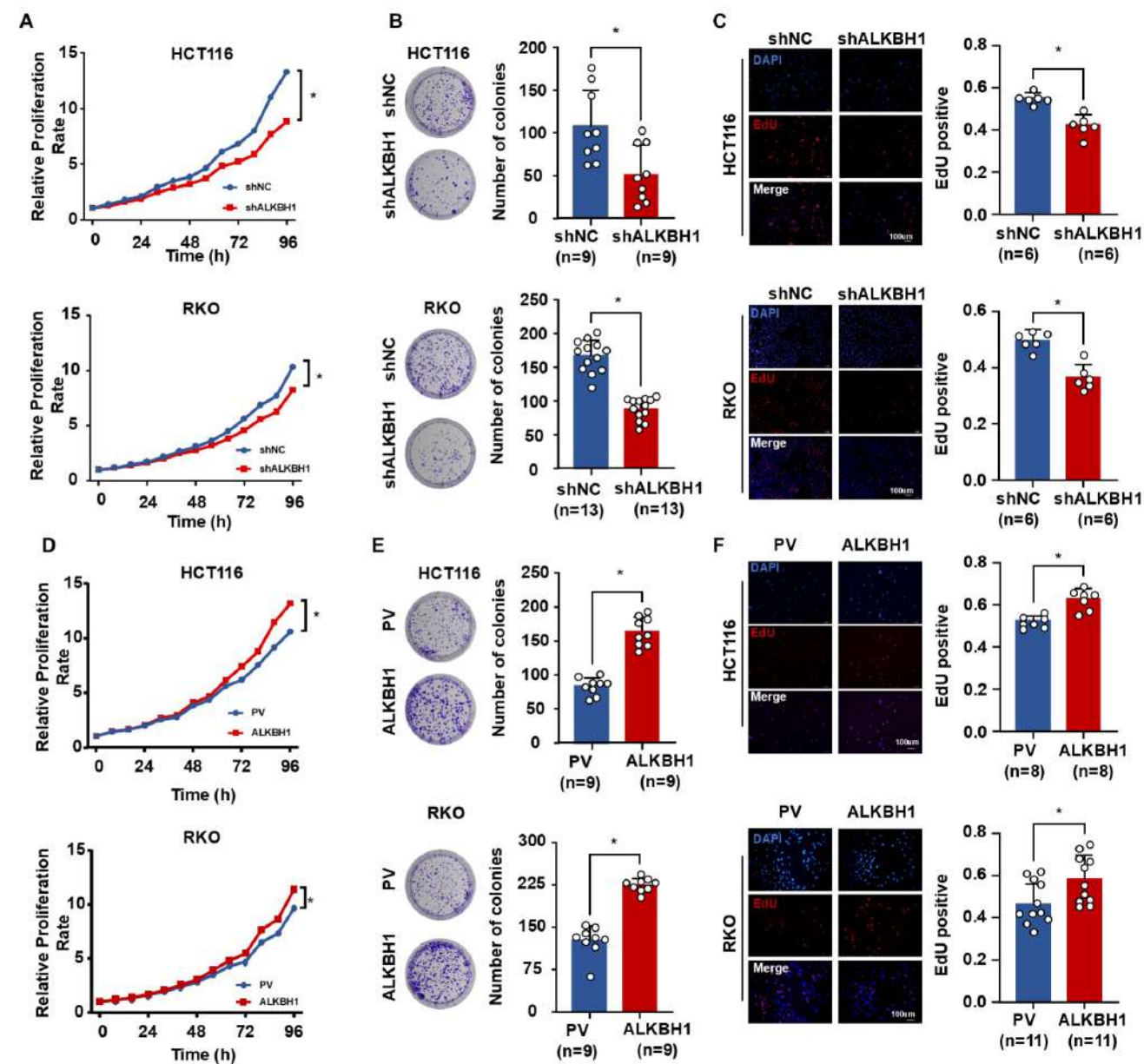

Fig. S8

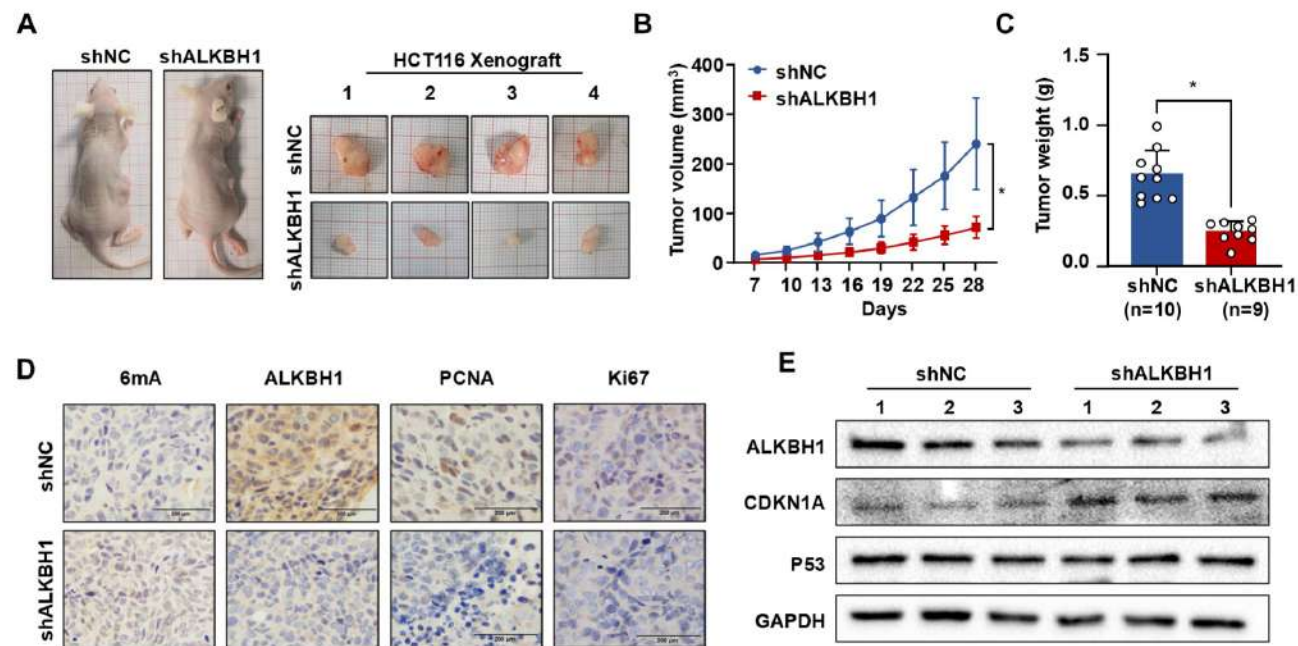

Fig. S9

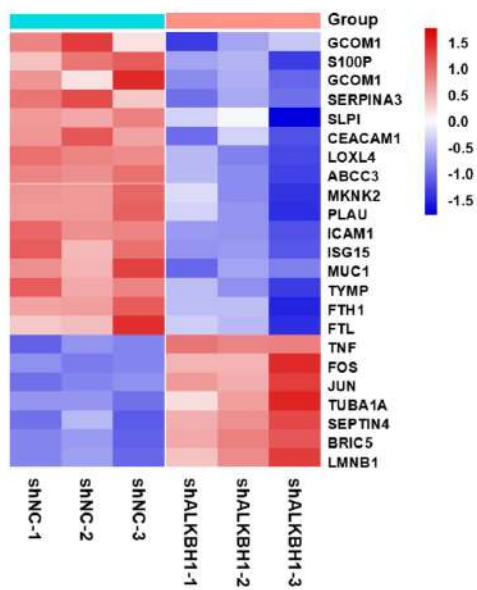

Fig. S10

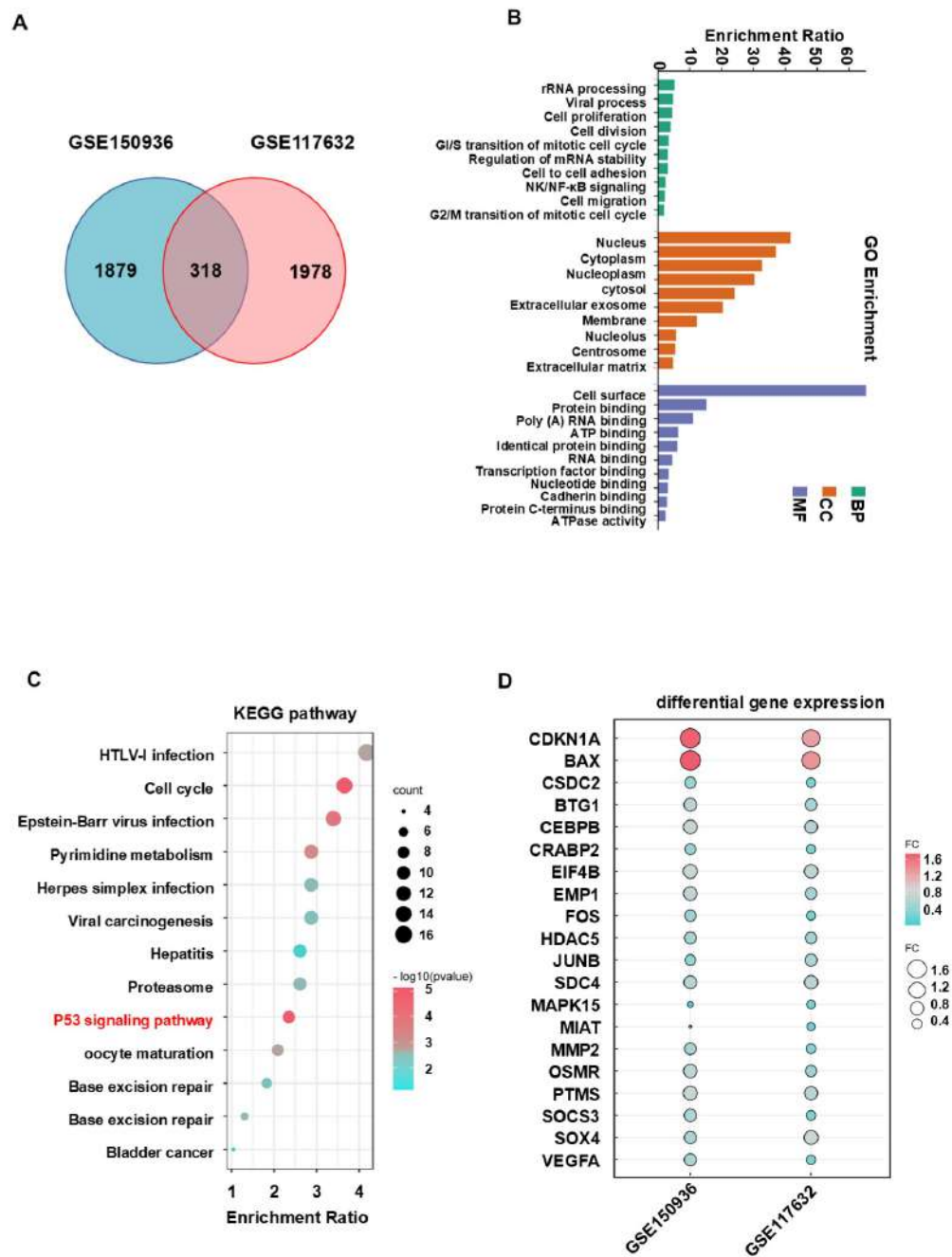

Supplement: Supplementary file 2 — Supplementary Material 2: Supplementary figures [file 40164_2025_704_MOESM2_ESM.pdf]
